# Genome-wide analysis of *Brucella melitensis* growth in spleen of infected mice allows rational selection of new vaccine candidates

**DOI:** 10.1371/journal.ppat.1012459

**Published:** 2024-08-26

**Authors:** Emeline Barbieux, Georges Potemberg, François-Xavier Stubbe, Audrey Fraikin, Katy Poncin, Angeline Reboul, Thomas Rouma, Amaia Zúñiga-Ripa, Xavier De Bolle, Eric Muraille

**Affiliations:** 1 Unité de Recherche en Biologie des Microorganismes (URBM)-Laboratoire d’Immunologie et de Microbiologie, NARILIS, University of Namur, Namur, Belgium; 2 Laboratoire de Parasitologie, and ULB Center for Research in Immunology (U-CRI), Université Libre de Bruxelles, Gosselies, Belgium; 3 Unité de recherche en physiologie moléculaire (URPhyM)-Laboratoire de Génétique moléculaire (GéMo), University of Namur, Namur, Belgium; 4 Departamento de Microbiología y Parasitología - IDISNA, Universidad de Navarra, Pamplona, Spain; Johns Hopkins University School of Medicine, UNITED STATES OF AMERICA

## Abstract

Live attenuated vaccines (LAVs) whose virulence would be controlled at the tissue level could be a crucial tool to effectively fight intracellular bacterial pathogens, because they would optimize the induction of protective immune memory while avoiding the long-term persistence of vaccine strains in the host. Rational development of these new LAVs implies developing an exhaustive map of the bacterial virulence genes according to the host organs implicated. We report here the use of transposon sequencing to compare the bacterial genes involved in the multiplication of *Brucella melitensis*, a major causative agent of brucellosis, in the lungs and spleens of C57BL/6 infected mice. We found 257 and 135 genes predicted to be essential for *B*. *melitensis* multiplication in the spleen and lung, respectively, with 87 genes common to both organs. We selected genes whose deletion is predicted to produce moderate or severe attenuation in the spleen, the main known reservoir of *Brucella*, and compared deletion mutants for these genes for their ability to protect mice against challenge with a virulent strain of *B*. *melitensis*. The protective efficacy of a deletion mutant for the *plsC* gene, implicated in phospholipid biosynthesis, is similar to that of the reference Rev.1 vaccine but with a shorter persistence in the spleen. Our results demonstrate that *B*. *melitensis* faces different selective pressures depending on the organ and underscore the effectiveness of functional genome mapping for the design of new safer LAV candidates.

## Author summary

Brucellosis is one of the most widespread bacterial zoonoses worldwide. We report here the use of transposon sequencing to compare the bacterial genes involved in the multiplication of *Brucella melitensis*, a major causative agent of brucellosis, in the lungs and spleens of infected mice. We found 257 and 135 genes predicted to be essential for *B*. *melitensis* multiplication in the spleen and lung, respectively, with 87 genes common to both organs, which demonstrates that *B*. *melitensis* faces different selective pressures depending on the organ. Then, we selected genes whose deletion is predicted to produce moderate or severe attenuation in the spleen, the main known reservoir of *Brucella*, and compared deletion mutants for these genes for their ability to protect mice against challenge with a virulent strain of *B*. *melitensis*. We observed that the protective efficacy of a deletion mutant for the *plsC* gene, implicated in phospholipid biosynthesis, is similar to that of the reference Rev.1 vaccine but with a shorter persistence in the spleen. Our results demonstrate the effectiveness of functional genome mapping for the design of new safer live attenuated vaccine candidates.

## Introduction

*Brucellae* are small Gram-negative facultative intracellular bacteria belonging to the Rhizobiales order within the α2-proteobacteria subgroup [1,2]. They are the causative agent of brucellosis, a common bacterial zoonotic disease responsible for important economic losses and public health issues, particularly in low- and middle-income countries [3–6]. *B*. *melitensis* is the species most often involved in ovine and caprine brucellosis and is also the most pathogenic species for humans [7].

Due to the impact of this disease on public health and the damage that it causes to the livestock industry, much effort has been expended to control or eradicate brucellosis in cattle and small ruminants. Three commercially available live attenuated vaccines (LAVs) are used to control *Brucella* infection in domestic animals: *B*. *abortus* S19 and RB51 to prevent brucellosis in cattle, and *B*. *melitensis* Rev.1 to protect sheep and goats. While the effectiveness of RB51 is controversial [8], S19 and Rev1 have been used in livestock wherever eradication has been successful. However, both vaccines have serious drawbacks [8–11]. Their attenuation was obtained empirically, and even accidentally for S19, which makes their virulence quite unpredictable. In some cases, they persist for years in the vaccinated host, where they can induce abortions and excretion in milk in animals, posing a high risk to humans for whom they are virulent [12]. Since in low- and middle-income regions mass vaccination is the only way to control the disease, which implies vaccination of pregnant animals, the control of brucellosis is therefore more complicated in these areas [13]. To encourage research in this area, the Bill and Melinda Gates foundation has offered a large prize to reward any new brucellosis vaccine that presents a safer profile.

The development of safer vaccines should involve functional mapping of the *Brucella* genome to identify the virulence genes that are essential at the different stages of *Brucella* infection in animals. This would allow for the rational development of a vaccine that persists just long enough in the host to induce frontline and systemic protective immune memory but does not remain long term in the reservoir organs.

Transposon sequencing (**Tn-seq**) is a powerful approach to rapidly and comprehensively determine an organism’s minimal genetic requirements for growth and survival under a variety of different conditions [14,15]. Previously, we performed various Tn-seq screens of *B*. *melitensis* 16M and identified a set of genes predicted as important for growth in 2YT nutrient rich media, *in vitro* in the RAW 264.7 macrophage cell line and *in vivo* in lungs from intranasally infected wild-type C57BL/6 mice [16]. Our main findings were that the genes that are essential for the multiplication of *B*. *melitensis in vitro* and *in vivo* are different and vary according to the immune status of the host.

Numerous observations suggest that the host is not a homogeneous environment for *Brucella*. In our mouse model, the early phase of the protective immune response against *B*. *melitensis* varies according to the organs: at 120 hours post-infection, an IL-17RA-dependent (Th17) response controls the infection in the lungs [17] and an IFNγ-dependent (Th1) response controls the infection in the spleen [18]. *B*. *melitensis* persists for more than 50 days in the spleen of infected mice but disappears after a few weeks from the lungs [19,20]. In addition, confocal microscopy analysis has demonstrated that *Brucella*-infected cells in the lung [16,21] and spleen [22,23] display a very different phenotype. Thus, we hypothesize that both the available nutrients as well as the microbicidal mechanisms encountered by *B*. *melitensis* in the lungs and spleen are different and that therefore a different set of bacterial genes might be required for the multiplication of *Brucella* in each organ. To test this hypothesis in this study we used Tn-seq screens to identify genes contributing to the fitness of *B*. *melitensis* 16M in spleen from wild-type and IFNγ-R^-/-^ C57BL/6 infected mice. By comparing these genes to those previously found in the lungs [16], we can select genes specifically necessary for the multiplication of *B*. *melitensis* in the spleen and test whether they can help us to develop a safer brucellosis vaccine.

## Results

### Tn-Seq identification of bacterial genes affecting the fitness of *B*. *melitensis* in the spleen of wild-type mice

As described previously [16], a *B*. *melitensis* 16M library of 3×10^6^ random mutants was constructed using a Kan^R^ derivative of the mini-Tn*5* transposon. This library was exposed to different selection conditions, such as culture in a 2YT rich medium for 24 hours, and in lungs from intranasally infected C57BL/6 mice for 120 hours. Following selection, surviving bacteria were collected and DNA was extracted and sequenced to identify mini-Tn5 insertion sites. For each selection condition, an average insertion index, called the **transposon insertion frequency** (**TnIF**), was associated with each gene of the *B*. *melitensis* genome. The TnIF is equal to the log_10_ (r+1) for a given coding sequence, with r = number of reads aligned in the central 80% of the coding sequence of the considered gene, as described in Materials and Methods.

The 2YT condition is the control condition (CTRL) for all our Tn-seq analyses of *B*. *melitensis* 16M, because whatever the condition analyzed, the bacteria are always cultured in 2YT after mice infection. Genes exhibiting a drop in TnIF of more than 0.5 compared to the average TnIF for the entire genome in the 2YT condition are considered to cause a significant attenuation of *B*. *melitensis* in 2YT. The threshold of 0.5 was determined based on the standard deviation of the bimodal TnIF distribution of the whole genome. It corresponds to 1.6–2.6 standard deviations, as described in the Materials and Methods. According to this criterion, 2460 of the 3369 genes of *B*. *melitensis* 16M are considered not to cause significant growth defect in the 2YT condition. Only these genes are analyzed under the other selection conditions.

**ΔTnIF** is calculated to quantify the attenuation of a gene in a condition other than 2YT. ΔTnIF = TnIF_cdt_−TnIF_CTRL_, where TnIF is computed for the test condition (TnIF_cdt_) and the control condition (TnIF_CTRL_).

In the present study, wild-type C57BL/6 mice were intraperitoneally infected with 5x10^6^ CFU of our *B*. *melitensis* 16M transposon mutant library and sacrificed at 120 hours post-infection, what corresponds to the peak of infection in the spleen [18]. As described previously [16], this high dose is necessary to administer the entire bank to each mouse and avoid bottleneck effects. Bacteria were extracted from this organ and analyzed as indicated in the Materials and Methods. The intraperitoneal route of infection was used in order to avoid the risk of a bottleneck which would lead to a sampling effect in the library. The TnIF values for each gene in the spleen condition were compared with those obtained previously [16] in the 2YT and lung conditions (S1 Table, page 1). A ΔTnIF for each gene in the lung and spleen conditions was calculated, as indicated previously and summarized in the Material and Methods section.

**Fig 1:**
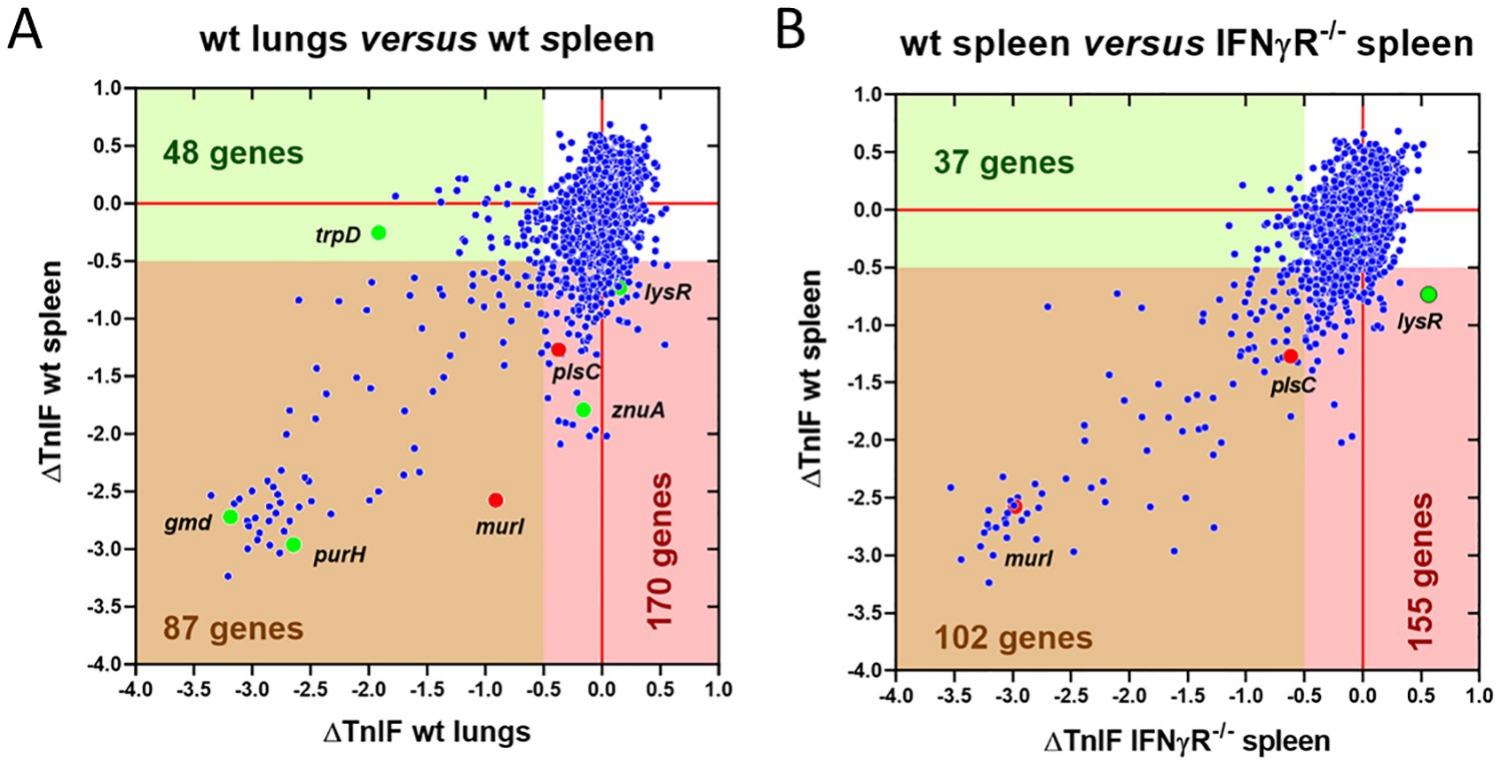
Comparison of *B*. *melitensis* genes required for optimal multiplication in the lung and the spleen conditions in wild-type and IFNγR^-/-^ mice. The figure shows the distribution of the ΔTnIF values of all *B*. *melitensis* genes (n = 2460) predicted not to induce an attenuation of fitness in 2YT rich medium (CTRL). Each gene is defined by two ΔTnIF values. (**A**) The x-axis indicates the ΔTnIF value for the lung of wt mice (TnIFlung—TnIF_CTRL_) and the y-axis indicates the ΔTnIF value for the spleen of wt mice (TnIF _spleen wt_—TnIF_CTRL_) at 120 hours post-infection. These ΔTnIF value comparisons show all the genes associated with a drop in fitness both in the lung and wt spleen (brown area), in the lung specifically (green area) or in the wt spleen specifically (pink area). (**B**) The x-axis indicates the ΔTnIF value for the spleen of IFNγR^-/-^ mice (TnIF _spleen IFNγR-/-_—TnIF_CTRL_) and the y-axis indicates the ΔTnIF value for the spleen of wt mice (TnIF _spleen wt_—TnIF_CTRL_) at 120 hours post-infection. These ΔTnIF value comparisons show all the genes restored in the IFNγR^-/-^ spleen (pink area).

A dot plot representation was used to visualize the differences between the lung and spleen conditions, where each dot corresponds to a *B*. *melitensis* gene defined by ΔTnIF values in the lung and spleen (Fig 1A). Among the 2460 genes analyzed (S1 Table, page 2), 257 genes are predicted, if inactivated, to cause a significant drop in *B*. *melitensis* fitness in the spleen condition and the same prediction was made for 135 genes in the lung condition (S1 Table, page 3 and 4, respectively). Only 87 genes are common to these two conditions (S1 Table, page 5), which implies that 170 genes are specific to the spleen condition (S1 Table, page 6) and 48 to the lung condition (S1 Table, page 7).

**Fig 2:**
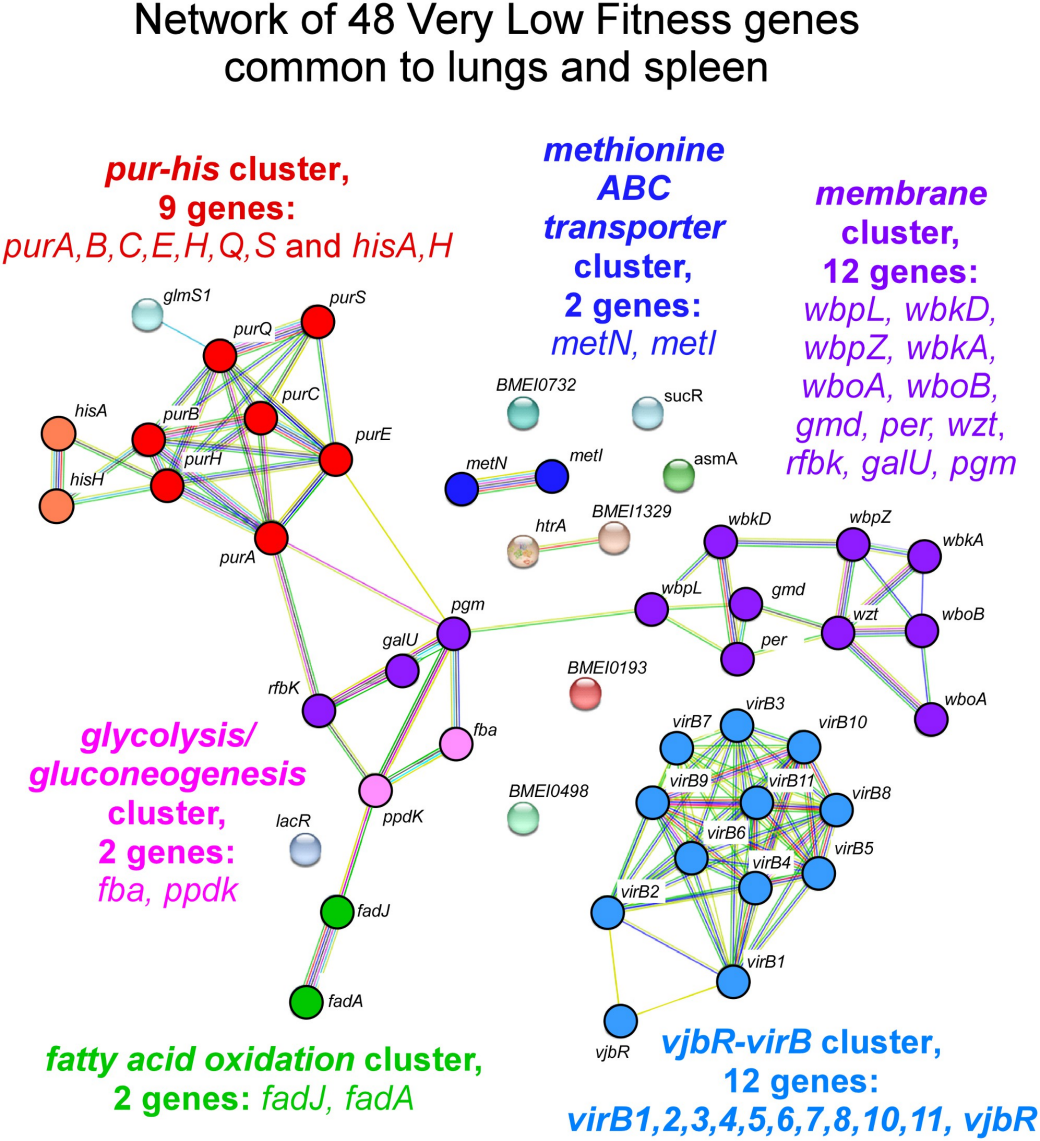
Clustering analysis of very low fitness genes common to the lung and spleen conditions at 120 hours post-infection. The diagram shows the potential interactions between the 48 genes displaying a ΔTnIF < -1.0 identified in the lungs and spleen of wild-type mice at 120 hours post-infection. The color code represents the different pathways. This clustering analysis was carried out with the STRING database (https://string-db.org).

A clustering analysis with the STRING database was carried out on the genes presenting a strong drop (-1.0) in ΔTnIF. The 48 genes displaying a ΔTnIF < -1.0 in both the lungs and the spleen included 6 gene clusters (Fig 2). These include a cluster of 9 genes involved in purine (*purA*,*B*,*C*,*E*,*H*,*Q*,*S*) and histidine (*hisA*,*H*) synthesis, a cluster of 2 genes associated with methionine transport (*metN*,*I*), a cluster of 2 genes involved in central carbohydrate metabolism (*fba*, *ppdK*), a cluster of 2 genes involved in fatty acid oxidation (*fadA*,*J*), a cluster of 12 genes necessary for the synthesis of the lipopolysaccharide (LPS) (*galU*, *gmd*, *per*, *pgm*, *manB*_*core*_*/rfbK*, *wboA*, *wboB*, *wbkD*, *wbpZ/wbkE*, *wbkA*, *wbpL/wbkF*, *wzt*) and a cluster of 12 genes forming the *virB* operon (*vjbR*, *virB1-11*) and encoding the type IV secretion system VirB and its transcriptional regulator VjbR [24,25].

**Fig 3:**
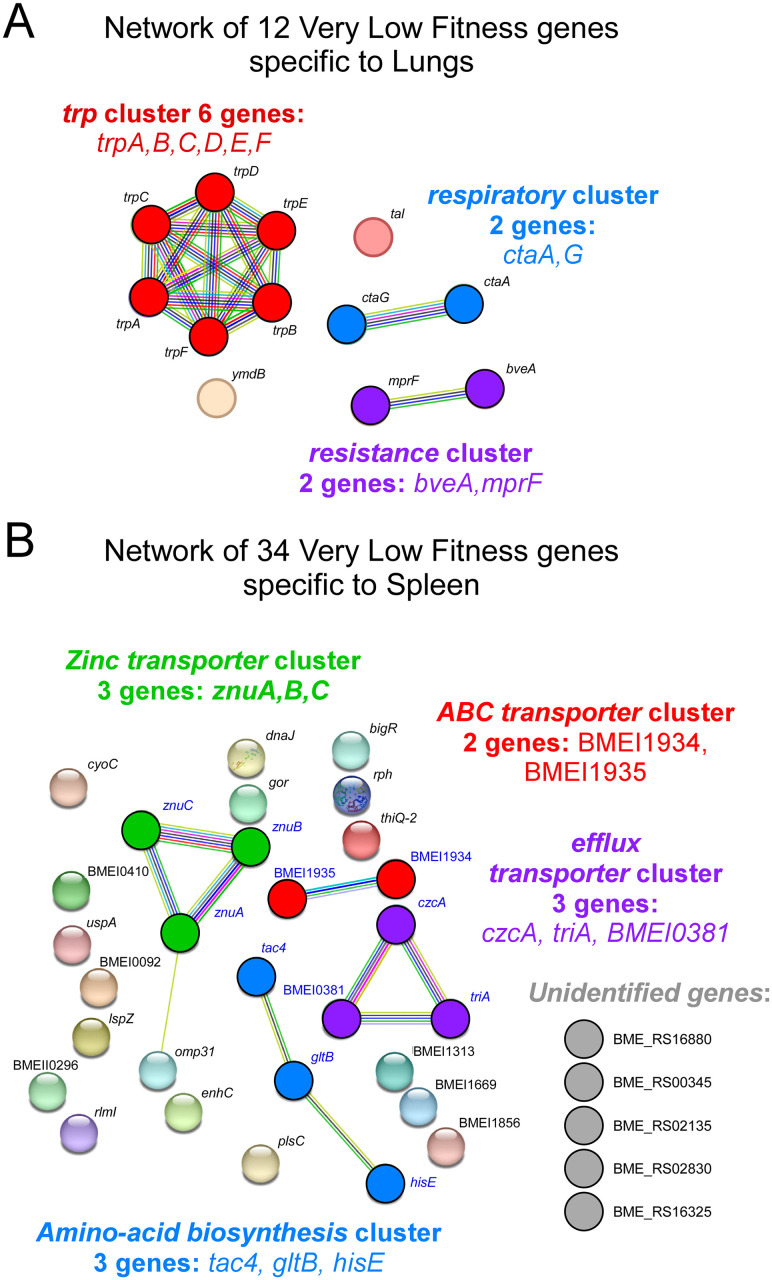
Clustering analysis of very low fitness genes specifically in the lung or spleen condition in wild-type mice at 120 hours post-infection. The diagram shows the potential interactions between genes displaying a ΔTnIF < -1.0 identified in the lungs or spleen of wild-type mice at 120 hours post-infection (12 genes in the lungs (**A**) and 34 genes in the spleen (**B**)). The color code represents the different pathways. This clustering analysis was carried out with the STRING database (https://string-db.org).

Among the 12 genes displaying a ΔTnIF < -1.0 in the lungs and > -0.5 in the spleen, a clustering analysis identified a cluster of 6 genes (*trpA*,*B*,*C*,*D*,*E*,*F*) involved in tryptophan synthesis, a cluster of 2 genes (*bveA*, *mprF*) associated with polymyxin resistance and a cluster of 2 genes (*ctaA*,*G*) associated with respiration that are indispensable in the lungs and not in the spleen (Fig 3A).

Regarding the genes displaying a ΔTnIF < -1.0 in the spleen and > -0.5 in the lungs, we identified 4 small clusters (Fig 3B). A cluster of 3 genes (*tac4*, *gltB*, *hisE*) associated with amino acid biosynthesis, a cluster of 3 genes (*znuA*,*B*,*C*) encoding a zinc transporter, a cluster of 3 genes (*czcA*, *triA*, BMEI0381) identified as an efflux transporter and a cluster of 2 genes (BMEI1934, BMEI1935) identified as an ABC transporter. Other 23 genes could not be linked to a gene cluster and 5 of them were unidentified before this study.

**Fig 4:**
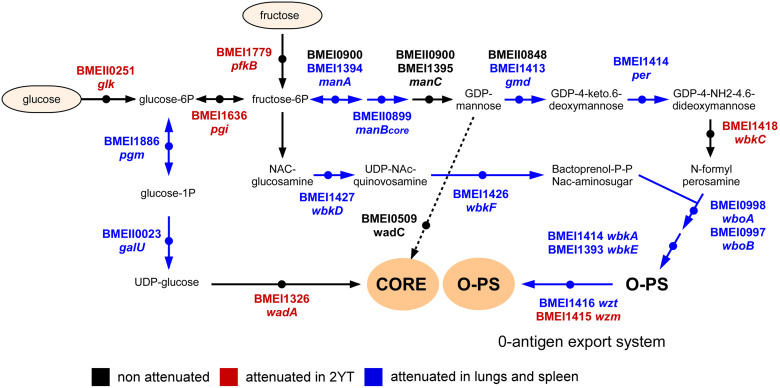
Identification of *B*. *melitensis* genes implicated in the lipopolysaccharide biosynthesis pathway required for optimal multiplication in the lungs and spleen of wild-type mice. Schematic representation of the lipopolysaccharide biosynthesis pathway of *B*. *melitensis* with genes attenuated in 2YT rich medium highlighted in red and genes required in the lung and spleen highlighted in blue (adapted from the KEGG PATHWAY database, https://www.genome.jp/kegg/pathway.html).

**Fig 5:**
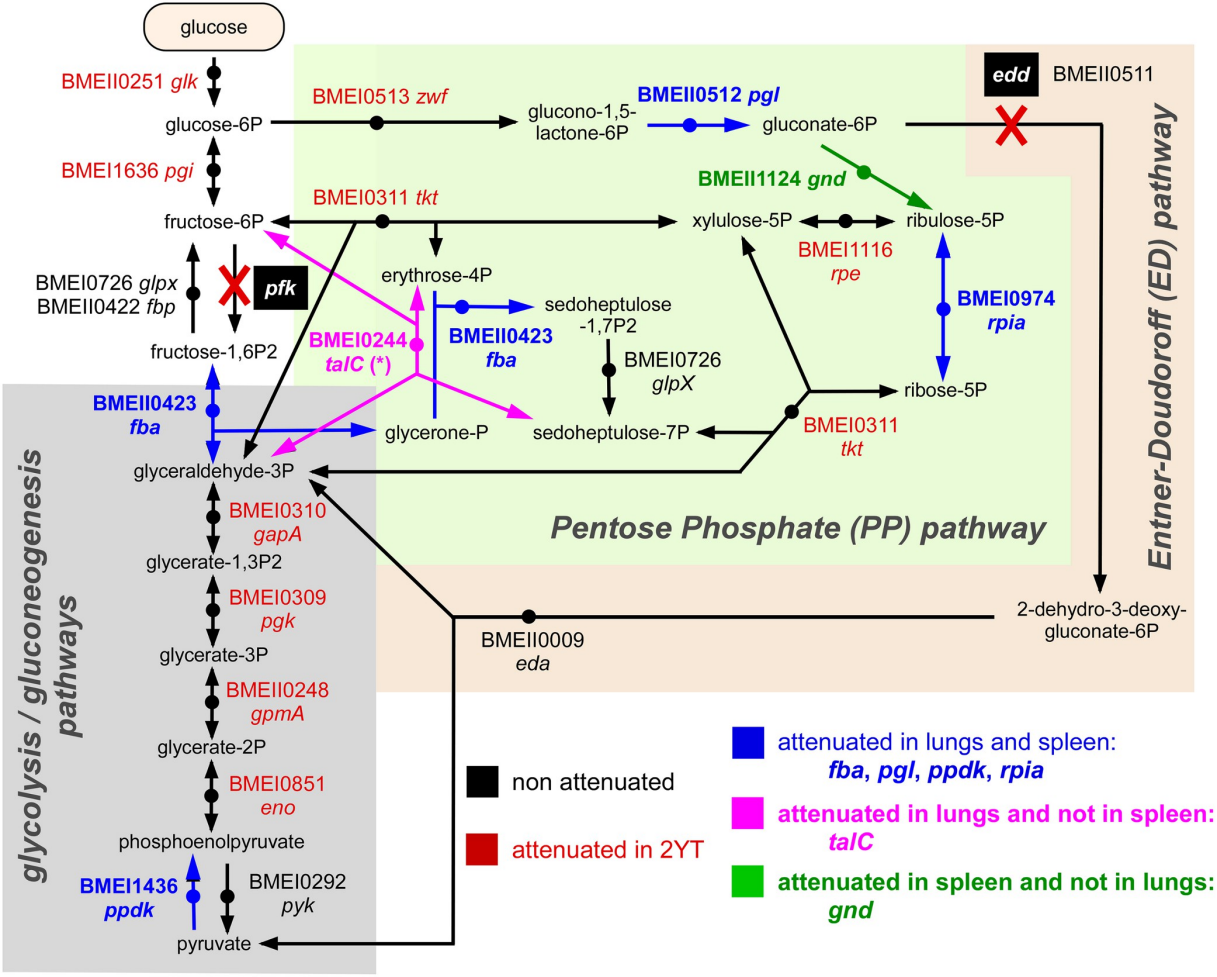
Identification of *B*. *melitensis* genes required in the central carbohydrate metabolism network for optimal multiplication in the lungs and spleen of wild-type mice. Schematic representation of the glycolysis, pentose phosphate and Entner-Doudoroff pathways in *B*. *melitensis* with genes attenuated in 2YT rich medium highlighted in red and genes attenuated in the lung and spleen, required only in the lung or only in the spleen respectively in blue, pink and green (adapted from the KEGG PATHWAY database, https://www.genome.jp/kegg/pathway.html).

**Fig 6:**
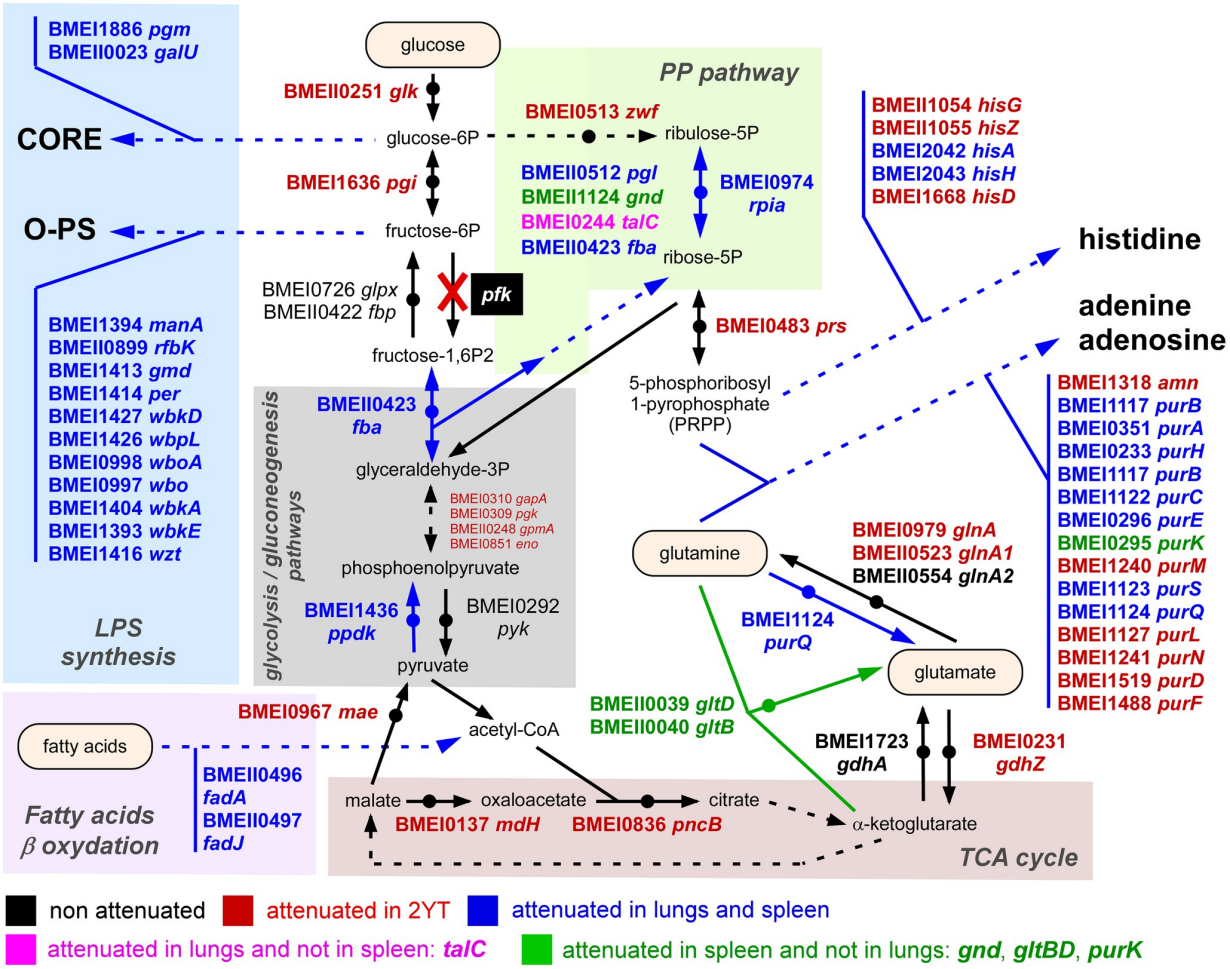
Integrated view of *B*. *melitensis* genes required in different metabolic pathways for optimal multiplication in lungs and spleen of wild-type mice. Schematic representation of lipopolysaccharide (LPS) synthesis, the pentose phosphate (PP) pathway, the fatty acid β oxidation, glucose (G) pathway and the tricarboxylic acid (TCA) cycle in *B*. *melitensis* with genes attenuated in 2YT rich medium highlighted in red and genes attenuated in the lung and spleen, required only in the lung or only in the spleen respectively in blue, pink and green (adapted from the KEGG PATHWAY database, https://www.genome.jp/kegg/pathway.html).

To summarize our Tn-seq data, we produced schematic representations of the LPS synthesis pathway (Fig 4) and of the central carbohydrate metabolism (Fig 5) and attempted to give a general view of the metabolism of *B*. *melitensis* by connecting these pathways to the tricarboxylic acid cycle, glutamine metabolism as well as the histidine and adenine/adenosine synthesis pathways (Fig 6). Interpretations are proposed in the Discussion section.

**Fig 7:**
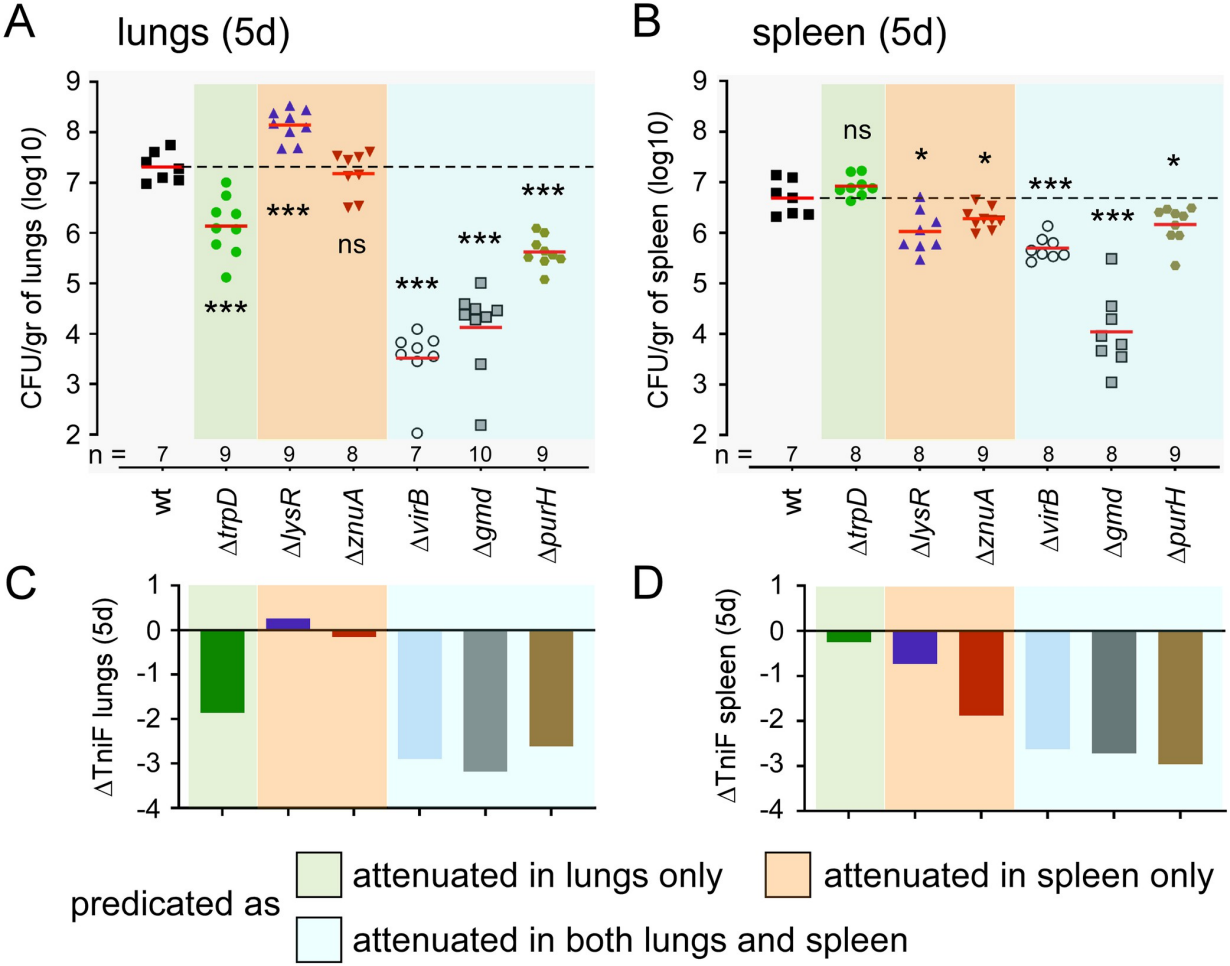
Functional confirmation of the prediction derived from the Tn-seq analysis of lung and spleen conditions in wild-type mice. Data shown in panels (**A**) and (**B**) are bacterial counts (CFU) at 120 hours post-infection in (**A**) lungs from wild-type mice infected intranasally and (**B**) the spleen from wild-type mice infected intraperitoneally with wild-type (wt), Δ*trpD*, Δ*lysR*, Δ*znuA*, Δ*virB*, Δ*gmd* and Δ*purH* strains of *B*. *melitensis* at the dose of 5x10^6^ CFU. Red lines represent the geometric mean. Dotted lines represent the mean of the wild-type condition. Significant differences between wt and the indicated groups are marked with asterisks: *p < 0.1, ***p < 0.001, in a (Wilcoxon-)Mann-Whitney post-test. These results are representative of three independent experiments. Data shown in panels (**C**) and (**D**) are the prediction derived from the Tn-Seq analysis expressed in ΔTnIF. ΔTnIF of genes in lungs from wild-type mice infected intranasally (TnIF lung—TnIF_CTRL_) are represented in (**C**). ΔTnIF of genes in the spleen from wild-type mice infected intraperitoneally (TnIF spleen—TnIF_CTRL_) are represented in (**D**). The color code of the legend indicates whether the genes are specifically attenuated in the lung (green), in the spleen (orange) or in both (blue) at 120 hours post-infection.

We attempted to confirm certain predictions from our Tn-seq analyses by producing deletion mutants of a few genes of interest. We selected *trpD* (BMEI0843), predicted to cause attenuation in the lungs; *znuA* (BMEII0178) and *lysR* (BMEI0513), predicted to cause attenuation in the spleen; and *gmd* (BMEI1426), *purH* (BMEI0233) as well as the *virB* operon genes, predicted to cause attenuation in both the lungs and the spleen. The wild-type (*wt*), Δ*gmd*, Δ*lysR*, Δ*purH*, Δ*trpD*, Δ*virB* and Δ*znuA* strains were intranasally administered at a dose of 5x10^6^ CFU to wild-type C57BL/6 mice and the number of bacteria present in the lungs at 120 hours post infection was measured by CFU counting (Fig 7A). The same strains were administered at the same dose by the intraperitoneal route to wild-type C57BL/6 mice and the number of CFUs in the spleen at 120 hours was determined (Fig 7B). The Tn-seq predictions for each of these genes in the lungs and spleen are shown in Fig 7C and 7D, respectively. We observed that the level of persistence of deletion mutants in the lungs and spleen was qualitatively well predicted by our Tn-Seq data. The Δ*gmd*, Δ*purH* and Δ*virB* strains were significantly attenuated compared to the wild-type strain in the lungs and spleen while the Δ*trpD* strain was attenuated only in the lungs and the Δ*lysR* and Δ*znuA* strains were only attenuated in the spleen. Overall, our data demonstrate that *B*. *melitensis* 16M is subject to different selection pressures in the lungs and spleen of C57BL/6 mice.

### Absence of the IFN-γR dependent signaling pathway affects the nature of essential bacterial genes in the spleen

Interferon-γ (IFN-γ) is well known to control *Brucella* growth *in vivo* as well as *Brucella*-induced inflammation [26]. Using the mouse model of intraperitoneal infection by *B*. *melitensis*, we have previously shown that production of IFN-γ in the spleen presents a peak at 120 hours post-infection and is mainly due to CD4^+^ T cells [27]. In principle, comparison of the genes that are essential for the growth of *B*. *melitensis* in the spleen of wild-type and IFN-γR^-/-^ mice should identify genes required for resistance to the IFN-γ-dependent immune response.

We infected IFN-γR^-/-^ C57BL/6 mice with 5x10^6^ CFU from our *B*. *melitensis* mini-Tn5 library. Mice were sacrificed at 120 hours post-infection, the spleen was collected, and bacteria were isolated and analyzed as described above. We point out that although the IFN-γ pathway is essential for the control of *B*. *melitensis* infection in the spleen [18], infected IFN-γR^*-/-*^ C57BL/6 mice do not yet show any clinical signs at this time of infection. We then compared the ΔTnIF for the wild-type and IFN-γR^-/-^ spleen conditions (S1 Table, page 2) for the 2460 genes which are not predicted to cause growth failure for *B*. *melitensis* in the 2YT condition using a dot plot presentation where each dot represents a gene (Fig 1B). Our results showed that among the 257 genes displaying a ΔTnIF < -0.5 in the wild-type spleen condition, only 102 displayed a ΔTnIF < -0.5 in the IFN-γR^-/-^ spleen condition. Thus, 155 *B*. *melitensis* genes predicted to cause attenuation in spleen are not predicted to cause attenuation in the spleen of IFNγR^-/-^ mice. Therefore, numerous genes seem to be involved in resistance to the IFN-γ-dependent immune response. S2 Table presents a list of the 19 genes presenting a ΔTnIF < -1.0 (very low fitness) in the wild-type spleen condition and a ΔTnIF > -0.5 (predicted not attenuated) in the IFNγR^-/-^ spleen condition. Unfortunately, a clustering analysis of these genes using the STRING database did not allow us to identify any cluster of genes, making it impossible to propose a hypothesis explaining how these genes are involved in the resistance of *B*. *melitensis* to the Th1 immune response.

**Fig 8:**
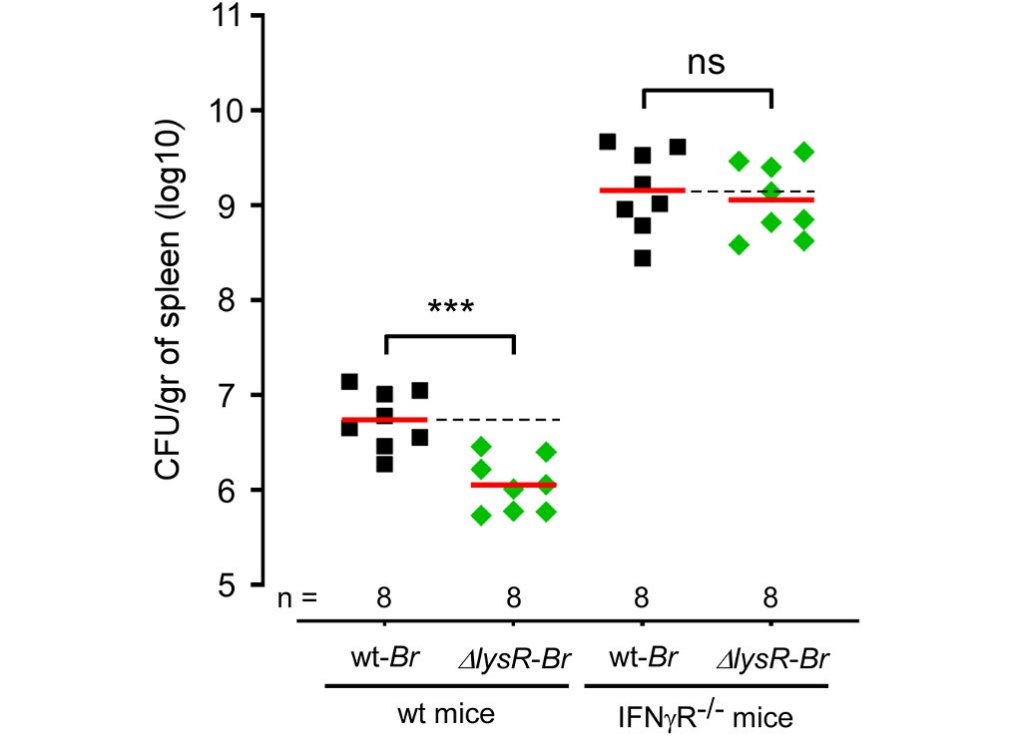
Functional confirmation of the prediction derived from the Tn-seq analysis of the spleen condition in wild-type and IFN-γR^*-/-*^ mice. Data shown are bacterial count s(CFU) at 120 hours post-infection in the spleen from wild-type and IFN-γR^-/-^ mice infected intraperitoneally with wild-type (wt) or Δ*lysR* strains of *B*. *melitensis* at a dose of 5x10^6^ CFU. Red lines represent the geometric mean. Dotted lines represent the mean of the wild-type condition. Significant differences between wt and the indicated groups are marked with asterisks: ***p < 0.001, in a (Wilcoxon-)Mann-Whitney post-test. These results are representative of two independent experiments.

In order to validate our Tn-seq predictions in the IFNγR^-/-^ spleen condition, we compared the persistence in the spleen of wild-type and IFN-γR^*-/-*^ mice of a deletion mutant of the *lysR* gene (BMEI0513, also called lysR21 and *vtlR* [28]). This gene displayed a ΔTnIF in the wild-type condition of -0.73 and a ΔTnIF of +0.56 in the IFNγR^-/-^ spleen condition. We infected wild-type and IFN-γR^-/-^ mice with 5x10^6^ CFU of *B*. *melitensis* 16M or the Δ*lysR* mutant. Evaluation of the number of bacteria at 120 hours post-infection confirmed the one log-attenuation of the Δ*lysR* strain in the spleen of wild-type mice while the mutant showed no attenuation in the spleen of IFN-γR^-/-^ mice (Fig 8). These results fully validate our Tn-seq predictions regarding the *lysR* gene.

### Selection of new vaccine candidates based on lung and spleen Tn-seq results

Ideally, to be protective, a brucellosis vaccine should multiply long enough in the lungs and spleen to induce mucosal and systemic immunity. Furthermore, an ideal vaccine should also not be able to persist in the spleen, which is the main known reservoir of *Brucella* in mice. To select genes whose deletion could lead to this type of behavior for *B*. *melitensis*, we should have Tn-seq data at different points of infection, for example at 5, 12 and days post infection. Unfortunately, in our model of *B*. *melitensis* infection, we only have Tn-seq data in the spleen up to 5 days post-infection. Beyond that period, the drop in the CFU count could lead to a bottleneck effect which causes sampling effects and makes the Tn-seq data uninterpretable. Therefore, we were not able to identify the *B*. *melitensis* genes necessary for a long-term chronic infection of the spleen. Accordingly, we decided to select genes whose inactivation is not expected to cause strong attenuation in the lungs, and which are expected to cause moderate or strong attenuation in the spleen at 120 hours post-infection. We hypothesized that genes thought to induce moderate attenuation at 120 hours in the spleen will cause stronger attenuation in the chronic phases of infection.

**Fig 9:**
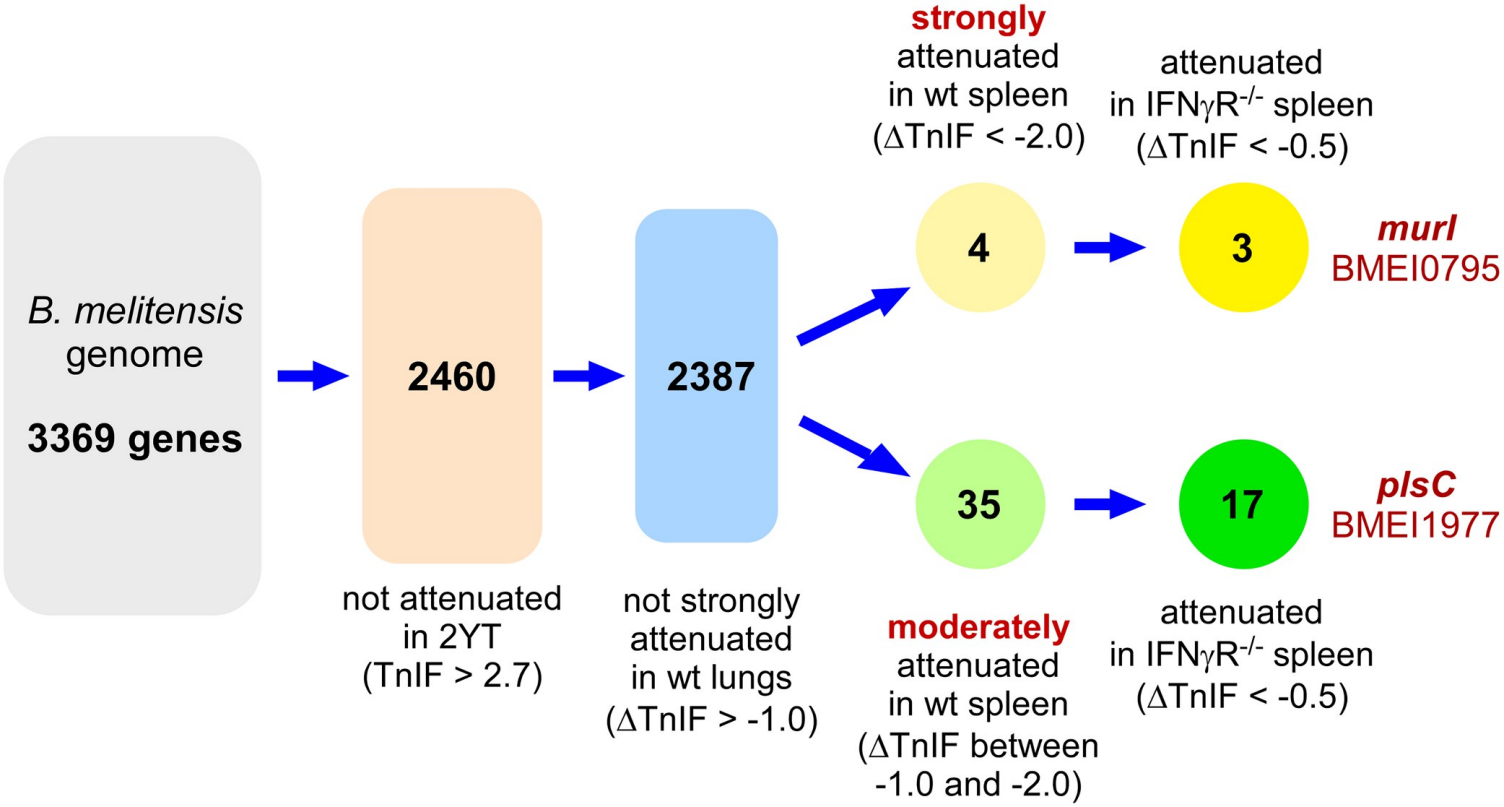
Tn-seq based strategy for selection of genes for development of vaccine candidates. We selected genes whose deletion could optimize a strain of *B*. *melitensis* to safely induce protective immunity. In practice, we selected the genes able to produce deletion mutants that induce a systemic immune response but do not persist long term in the spleen reservoir. Only genes that are not required to grow on 2YT rich medium were kept (ΔTnIF > 2.7) (2460 genes). In this group, genes that are not strongly attenuated (ΔTnIF > -1.0) in the lungs were conserved (2387 genes). Two different thresholds were then applied for the spleen condition in wild-type (wt) mice: either strongly attenuated (ΔTnIF < -2.0) or moderately attenuated (-2.0<ΔTnIF<-1.0). In addition, the candidate must not revert in immune deficient mice (IFNγR^-/-^) (ΔTnIF < -0.5). ΔTnIF corresponds to the TnIF tested condition—TnIF_CTRL_ (2YT rich medium).

Bearing the above in mind, from the 3369 genes of the *B*. *melitensis* genome we selected the 2460 genes that, when inactivated, were not predicted to cause an attenuation in 2YT medium (TnIF > 2.7 in 2YT condition) (Fig 9). Then, we selected the 2387 genes predicted not to cause strong attenuation in the lungs when inactivated (ΔTnIF > -1.0 in the lung condition). From this category we selected the genes whose deletion was predicted to cause a moderate (ΔTnIF between -1.0 and -2.0 in wild-type spleen condition) or a strong attenuation (ΔTnIF between < -2.0 in wild-type spleen condition) in the spleen (Fig 9). Finally, we verified that these candidates remain predicted as attenuated in IFNγR^-/-^ mice (ΔTnIF < -0.5 in the IFNγR^-/-^ spleen condition) to avoid a possible increase in virulence of the vaccine strain in the event of a deficient Th1 response. From the 3 genes predicted to cause strong attenuation in the spleen (S1 Table, page 7) we selected *murI* (BMEI0795) and from the 17 genes predicted to cause moderate attenuation (S1 Table, page 8) we selected *plsC* (BMEI1977). We selected these two genes because although the enzymatic functions of the encoding proteins are known, their role in *Brucella* pathogenesis has, to our knowledge, not yet been investigated.

**Fig 10:**
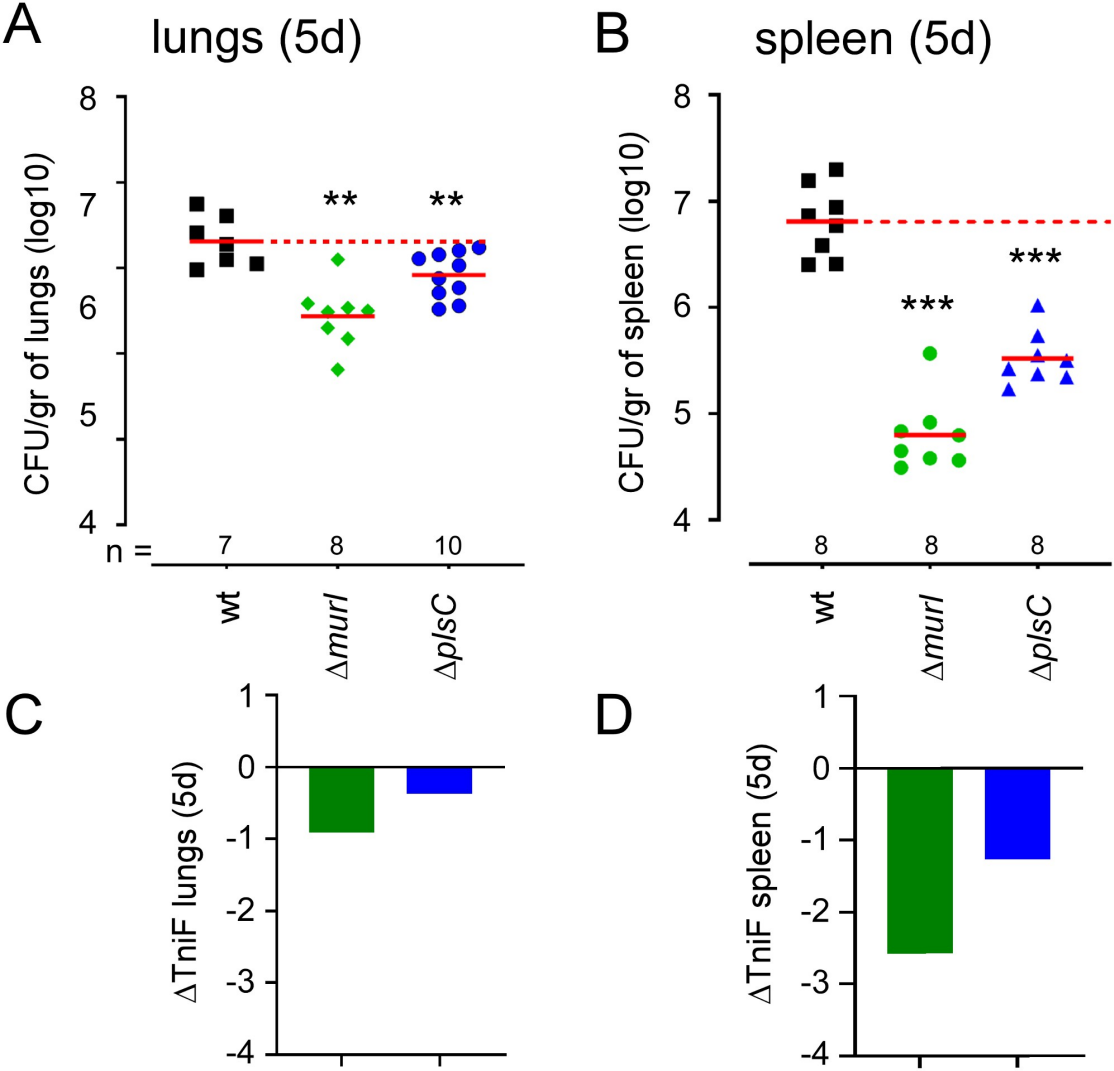
Functional confirmation of the prediction derived from Tn-seq analysis of the lung and spleen conditions in wild-type mice for the *murI* and *plsC* genes. Data shown in panels (**A**) and (**B**) are bacterial counts (CFU) at 120 hours post-infection in (**A**) lungs from wild-type mice infected intranasally and (**B**) the spleen from wild-type mice infected intraperitoneally with wild-type (wt), Δ*murI* and Δ*plsC* strains of *B*. *melitensis* at a dose of 5x10^6^ CFU. Red lines represent the geometric mean. Dotted lines represent the mean of the wild-type condition. Significant differences between wt and the indicated groups are marked with asterisks: *p < 0.1, **p < 0.01, ***p < 0.001, ****p < 0.0001, in a (Wilcoxon-)Mann-Whitney post-test. These results are representative of three independent experiments. Data shown in panels (**C**) and (**D**) are the prediction derived from the Tn-Seq analysis expressed in ΔTnIF of genes in the lungs (**C**) and spleen (**D**) from wild-type mice infected intraperitoneally (TnIF spleen—TnIF_CTRL_).

Tn-seq predictions for these two genes were verified by constructing deletion mutants and measuring their persistence in the spleen (Fig 10). For that purpose, wild-type C57BL/6 mice were infected intranasally or intraperitoneally with 5×10^6^ CFU of the wt, Δ*murI* and Δ*plsC* strains and sacrificed at 120 hours post-infection, and the numbers of bacteria was evaluated in the lungs and spleen by CFU-counting. Our observations confirmed that inactivation of both genes leads to low attenuation in the lungs (-1.0 and -0.5 log CFU, respectively) and that inactivation of *murI* and *plsC* induces strong (-2.0 log CFU) and moderate (-1.3 log CFU) attenuation in spleen, respectively.

To ensure that the observed phenotype was indeed consequence of the absence of the *murI* and *plsC* genes and not a non-specific effect caused by the deletion, we complemented these mutants with an intact coding sequence on a low copy plasmid, as indicated in the Material and Methods section. We observed that the complemented strains displayed a level of CFU in the lungs and spleen close to that of the wt strain (S1 Fig), demonstrating that the attenuation of the mutants was not due to off-target effects.

**Fig 11:**
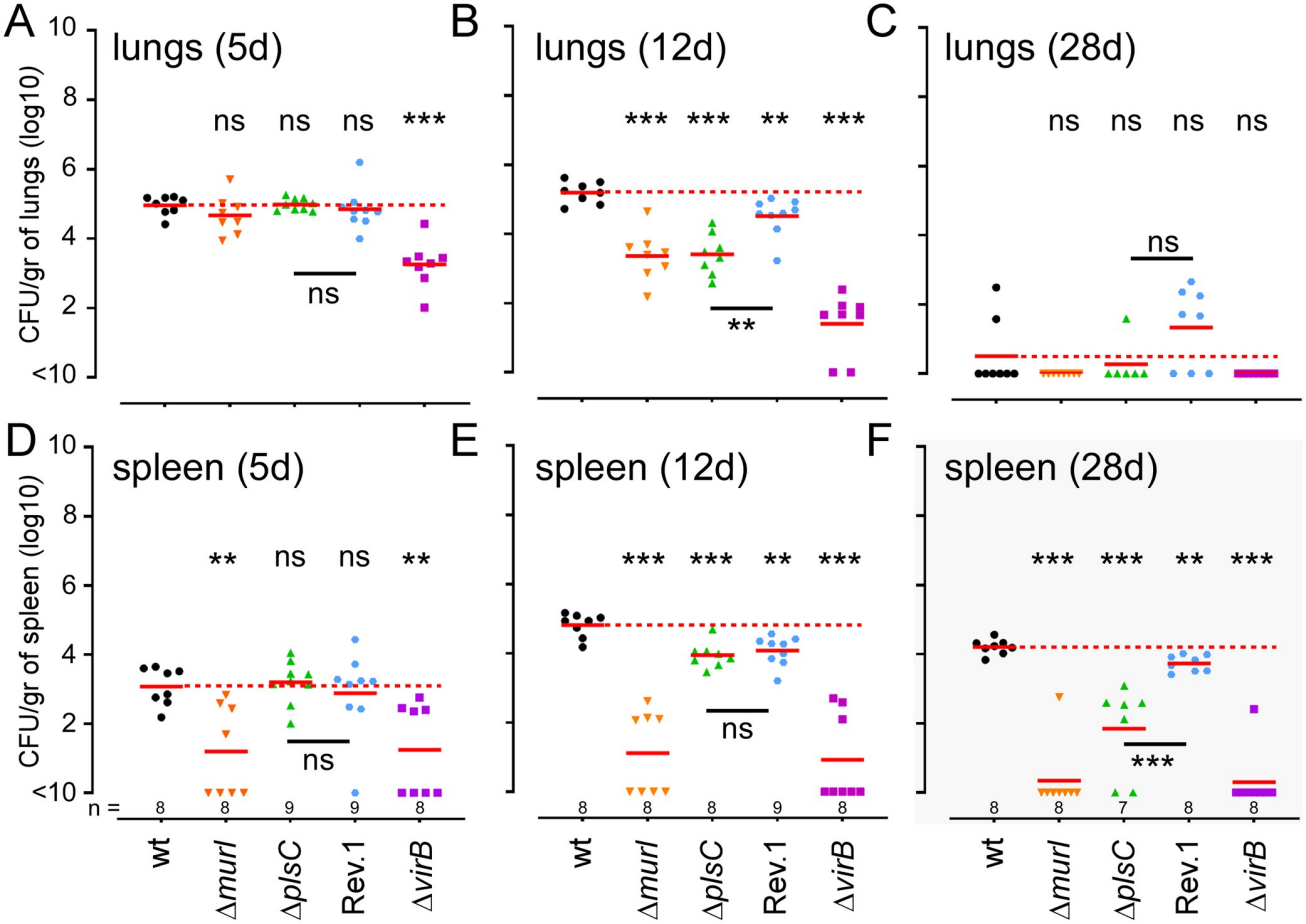
Comparison of long-term persistence of different vaccine candidates in the lungs and spleen. Wild-type C57BL/6 mice were infected intranasally with 10^5^ CFU of different *B*. *melitensis* strains: wild-type, Δ*murI*, Δ*plsC*, Rev.1 or Δ*virB*. This dose was used to mimic the infection following vaccination. Data shown are bacterial counts (CFU) at 5 (**A**), 12 (**B**) and 28 (**C**) days post-infection in the lung and at 5 (**D**), 12 (**E**) and 28 (**F**) days post-infection in the spleen. Red lines represent the geometric mean. Dotted red lines represents the mean of the wild-type strain. Significant differences between wt and the indicated groups are marked with asterisks: *p < 0.1, **p < 0.01, ***p < 0.001, ****p < 0.0001, in a (Wilcoxon-)Mann-Whitney post-test. These results are representative of three independent experiments.

Finally, as we planned to test the protective capacity of these vaccine candidates in the intranasal vaccination model with a dose of 10^5^ CFU characterized previously [17,20], we also tested the persistence of the Δ*murI* and Δ*plsC* strains in the lungs and the spleen under these conditions. Wild-type C57BL/6 mice were infected intranasally with 10^5^ CFU of the wt, Δ*virB*, Δ*murI* and Δ*plsC* strains as well as with the Rev.1 vaccine. The Δ*vir*B strain was used as a negative control due to its low persistence in the lungs and spleen. Comparison of our mutant strains with the Rev.1 vaccine will allow us to determine whether our vaccines persist less in animals than the reference vaccine and therefore whether we can expect these vaccine candidates to be safer than Rev.1. As expected, we observed (Fig 11) that the Δ*virB* strain was rapidly eliminated from the lungs and did not colonize the spleen. The Δ*murI* strain persisted for up to 12 days in the lungs but was unable to colonize the spleen. In contrast, the Δ*plsC* strain persisted in the lungs at the same level as the Δ*murI* strain but succeeded in colonizing the spleen. At 12 days, the level of bacteria in the spleen from the Δ*plsC* strain and Rev.1 were close to that associated with the wt strain. However, Δ*plsC* bacterial burden dropped drastically at 28 days post-infection while the wt strain and Rev.1 persisted in the spleen. Under our vaccination conditions, the Δ*murI* strain is therefore able to multiply in the lungs but is not able to colonize the spleen, whereas the Δ*plsC* strain can temporarily colonize the spleen but is unable to establish itself there over the long term.

### Evaluation of the protective capacity of the Δ*murI* and Δ*plsC* vaccine candidates

**Fig 12:**
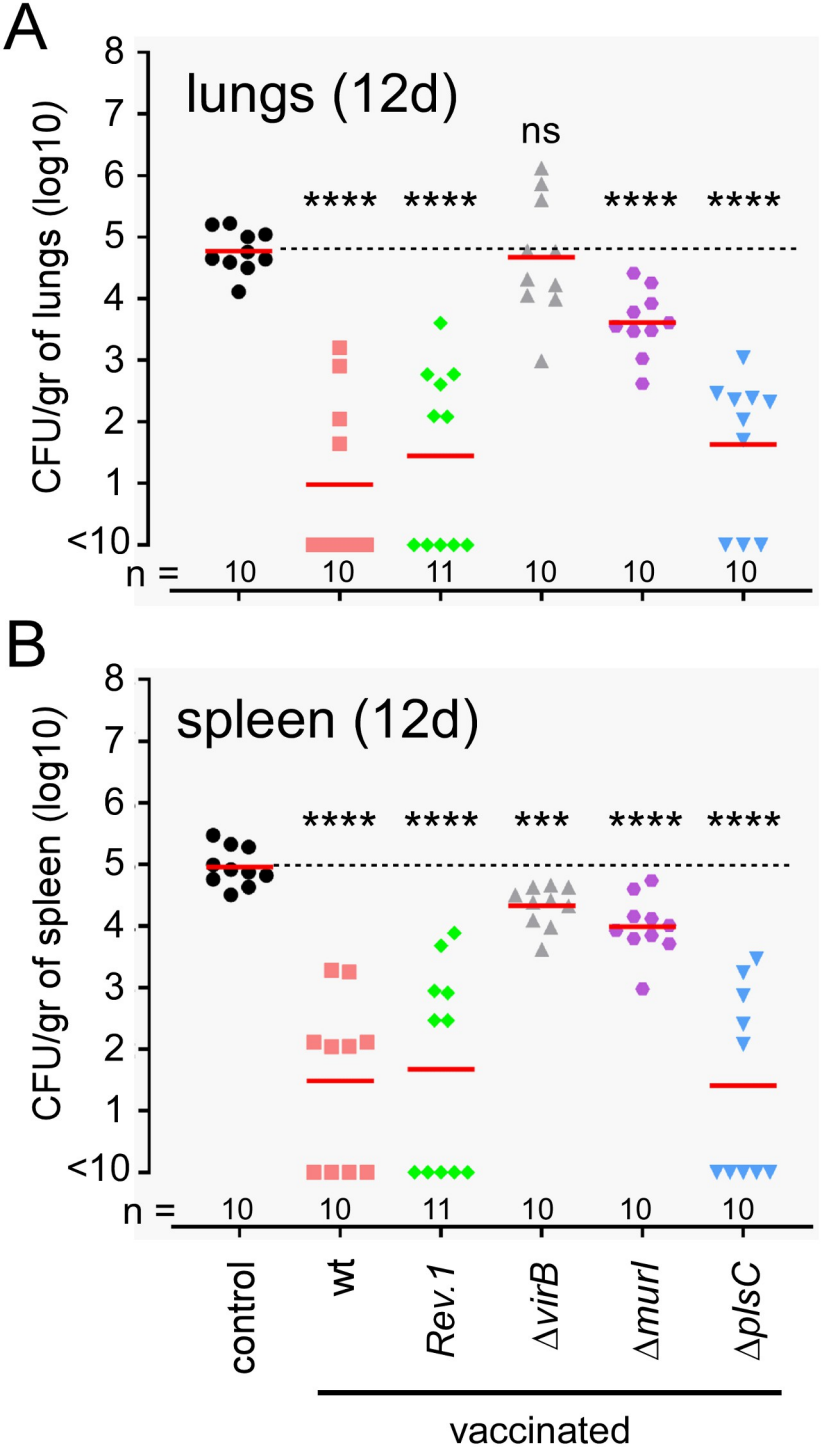
Evaluation of the protective capacity of Δ*murI* and Δ*plsC* vaccine candidates. Wild-type C57BL/6 mice were immunized intranasally with 10^5^ CFU of different *B*. *melitensis* strains: wild-type, Rev.1, Δ*virB*, Δ*murI* or Δ*plsC*. Wild-type *B*. *melitensis* 16M strain and the Rev.1 vaccine were used as positive controls. Δ*virB* was used as the negative control, as it is known to be rapidly eliminated from the body and thus does not have time to induce a memory response. Δ*murI* and Δ*plsC* were the selected vaccine candidates. Unvaccinated (control) and vaccinated wild-type mice were challenged intranasally at 6 weeks post-vaccination with 10^*5*^ CFU of wild-type mCherry–*B*. *melitensis* and sacrificed at 12 days post-infection. The data represent the number of CFU/g in the lung (**A**) and spleen (**B**). Red lines represent the geometric mean. Dotted lines represent the mean of CFU for unvaccinated mice. Significant differences between the control (unvaccinated) and groups of vaccinated mice are marked with asterisks: ***p < 0.001, ****p < 0.0001, in a (Wilcoxon-)Mann-Whitney post-test. These results are representative of three independent experiments.

In order to assess the ability of the Δ*murI* and Δ*plsC* vaccine candidates to induce a long-lasting protective memory, wild-type C57BL/6 mice intranasally received 10^5^ CFU of *B*. *melitensis* wt, Rev.1, Δ*virB*, Δ*murI* and Δ*plsC* strains and were challenged 6 weeks later via the same route with 10^5^ CFU of the virulent *wt* strain, as described in the Materials and Methods. This challenge strain expressed a fluorochrome and resistance to kanamycin in order to be differentiated from the vaccination strains. One group of mice (control) was not vaccinated. As expected, we observed that the wt and Rev.1 strains induced a very effective immune response that reduced the challenge strain load in the lungs and spleen by 3 log CFU (Fig 12). In contrast, the Δ*virB* strain induced no or very weak detectable protective immunity in the lungs and spleen (-0.5 log CFU), respectively. Finally, Δ*murI* induced only weak immunity in the lungs and spleen (-1 log CFU) whereas Δ*plsC* induced a protective immune response similar to the wild-type and Rev.1 strains.

Interestingly, these results could be correlated with the induction of specific *Brucella* IgG2a antibodies since protective strains (*wt*, Rev.1 and Δ*plsC*) induced high levels of antibodies while non-protective strains (Δ*virB*, Δ*murI*) did not (S2 Fig).

## Discussion

Brucellosis is one of the most common bacterial zoonoses and has a significant economic and public health impact worldwide. *Brucella* infections cause abortions and sterility in ruminants and pigs and a severely debilitating febrile illness in humans [3–5], an accidental host that become infected through contact with infected animals or by consuming contaminated animal products. Since cases of human-to-human transmission are extremely rare, efforts to control brucellosis should first focus on farm animals. Control of brucellosis in northern European countries and the USA was possible through a policy combining mass vaccination and slaughter of infected herds, a strategy that is difficult to apply in many southern countries, like African countries, where the state cannot compensate breeders for the slaughter of herds or carry out sufficiently rigorous screening for infected animals. In these countries, the fight against brucellosis relies heavily on vaccines. Unfortunately, the only vaccines that are effective on the ground are live attenuated vaccines and those currently recommended, such as S19, RB51 and Rev.1, have several important shortcomings [9–11].

*B*. *melitensis*, which mainly infects goats and sheep, is the most pathogenic *Brucella* species for humans [7]. The live attenuated *B*. *melitensis* Rev.1 strain is recommended by the World Organization for Animal Health (WOAH) for vaccination of small ruminants since conjunctival Rev.1 administration successfully protects these animals [29]. However, this vaccine has several important drawbacks [9,30]. Rev.1 is a natural revertant of a virulent *B*. *melitensis* 6056 strain rendered dependent on streptomycin in the mid-1950s, one of the antibiotics recommended for the treatment of human brucellosis. Also, Rev.1 is a smooth strain and as such induces positive serology which interferes with the diagnosis of brucellosis, it causes dose dependent abortions in small ruminants [9,31], it can persist for a long time in the vaccinated animal and be excreted in the milk [32,33] and it is virulent for humans [34,35]. Comparative proteomic analysis of the virulent *B*. *melitensis* 16M strain with the attenuated Rev.1 strain in an acid medium *in vitro* showed that the two strains presented important differences [36]. At least 403 genes involved in complex cellular processes, like metabolism and transport, are expressed at different levels making very complicated to identify the mechanisms of the attenuation of Rev.1.

In this study, we tested the rational development of new attenuated vaccine candidates from the virulent *B*. *melitensis* 16M strain based on a functional map of its genome produced by Tn-Seq. We have identified the genes that are essential for the multiplication of *B*. *melitensis* 16M in the spleen from wild-type and IFNγR^-/-^ C57BL/6 mice and compared them with the genes previously identified by our team as essential for multiplication in the lungs of C57BL/6 mice [16]. This comparison enabled us to identify the genes essential for the multiplication of *B*. *melitensis* in both the lungs and spleen as well as the genes specifically necessary for multiplication in the lungs or spleen. The predictions from our Tn-Seq analyses were validated by constructing deletion mutants for the genes of interest and measuring their persistence in the lungs and spleen of infected mice.

We collected a very large amount of information that will be of interest for future aspects of research on *B*. *melitensis*. Here, we attempt to summarize the major trends in *B*. *melitensis* metabolism in mice suggested by the analysis of our Tn-Seq data.

A clustering analysis of the 48 genes predicted to be essential in both the lungs and spleen identified 6 clusters involved in purine and histidine biosynthesis, methionine transport, carbohydrate metabolism, fatty acid oxidation, LPS biosynthesis and the type IV secretion system encoded by the *virB* operon (Fig 2). These data confirm the importance of bacterial genes implicated in LPS synthesis for *B*. *melitensis* survival *in vivo* [37]. While most of the genes involved in the biosynthesis of the LPS from glucose and fructose are not essential for *B*. *melitensis* 16M to multiply in rich medium or in the macrophage line RAW 264.7 [16], they become essential in the lungs [16] and spleen (Fig 4). This is consistent with the fact that *B*. *melitensis* is extracellular in the early times of intranasal [16] and intraperitoneal infection [38] and therefore exposed to attack by the innate immune system. It is well demonstrated *in vitro* that rough strains (LPS O-chain deficient) of *Brucella* are more sensitive to complement [39,40] and bactericidal cationic peptides [41]. Our data also confirm previous works documenting the importance of genes implicated in methionine transport [42], purine metabolism [43] and genes of the *virB* operon [44], with the exception of *virB12* [45] for *B*. *abortus* growth in mice.

Regarding central carbohydrate metabolism, bacteria might present these interconnected pathways: classical glycolysis/gluconeogenesis, the pentose phosphate (PP) pathway, the Entner–Doudoroff (ED) pathway and the citric acid cycle (TCA). Numerous *in vitro* studies (reviewed in [46,47]) have shown that *Brucella* present a complete TCA cycle but are naturally deficient in the enzyme *pfk* and in consequence, in glycolysis. Therefore, the attenuation generated by *fba* (BMEII0423) deletion suggests an important role for gluconeogenesis during *Brucella* multiplication in lungs and spleen (Fig 5), results in accordance with the observations made by other authors [48,49] In this regard, inactivation of the *pyk* gene (BMEI0292), which converts phosphoenolpyruvate (PEP) to pyruvate, does not lead to *B*. *melitensis* attenuation in any of the conditions tested, while the enzyme PpdK (BMEI1436), which converts pyruvate to PEP, is essential *in vivo* (Fig 6). In addition to a possible role of PpdK in gluconeogenesis, the produced PEP is necessary to synthesize phenylalanine, tyrosine, tryptophan, glycerolipids, and other PEP-derived molecules [49].

Concerning the ED pathway, the first enzyme of the route, Edd (BMEII0511), is inactive in *B*. *abortus* and *B*. *melitensis* due to a nonsynonymous mutation [47], and therefore, these two strains exclusively use the PP pathway for hexose catabolism. In keeping with this observation, we found that two key enzymes of the PP pathway become indispensable for *B*. *melitensis* 16M multiplication in the lungs and spleen: *pgl* (BMEII0512) and *rpiA* (BMEI0974) (Fig 5). We also observed that many enzymes involved in the synthesis of histidine (*hisA*,*H*) and adenine (*purA*,*B*,*C*,*E*,*H*,*S*,*S*) from 5-phosphoribosyl 1-pyrophosphate produced by the PP pathway become indispensable *in vivo* (Fig 6).

The Tn-seq data suggest that fatty acid catabolism might be important during infection of the lungs and spleen since *fadA* (BMEII0496) and *fadJ* (BMEII0497), putatively required for fatty acid utilization, play an essential role during the multiplication of *B*. *melitensis* 16M in these organs (Fig 6). In this regard, the glyoxylate shunt is essential for utilizing acetate and fatty acids as carbon sources under physiological conditions requiring gluconeogenesis [50] However, the two enzymes of this cycle, isocitrate lyase *aceA* (BMEI0409) and malate synthase *glcB* (BMEI0380), are not predicted to be essential *in vivo*, data in agreement with previous results in *B*. *abortus* [49] and *B*. *suis* [48,51]

Also, the Tn-seq data predict that the glutamate synthase (GOGAT) enzyme, formed by *gltB* (BMEII0040) and *gltD* (BMEII0039), is essential during *B*. *melitensis* 16M multiplication in the spleen. GS-GOGAT is a two reaction-cycle that incorporates ammonium into glutamate to obtain glutamine (glutamine synthetase, GS), followed by the transfer of the amide group into a-ketoglutarate by GOGAT, with an overall yield of one glutamate molecule. In line with these results, mutants in *gltB* and *gltD* are attenuated both in cells and mice [52,53] These results might indicate that, although glutamine/glutamate are critical because they serve as the N donor for the biosynthesis of most amino acids, amino sugars, purines, pyrimidines, NAD+ and p-aminobenzoate [54], they are not provided in sufficient amounts in the infected cells of the spleen.

Collectively, these Tn-seq data suggest that *B*. *melitensis* could face multiple nutritional stresses in mice, which is consistent with its intracellular localization in activated phagocytic cells. *In vivo*, *B*. *melitensis* seems to have to synthesize histidine and adenine itself and import methionine. It also seems to be dealing with glucose deprivation. In keeping with these hypotheses, activation of the MyD88 pathway in macrophages has been shown to promote M1 metabolic polarization favoring glycolysis and glucose consumption able to directly restrict *Brucella* proliferation [55]. Thus, in an M1 polarized intracellular environment low in glucose, we hypothesize that *B*. *melitensis* would use amino acid such as glutamate and glutamine as well as fatty acids from the host cells to allow LPS biosynthesis and production of essential amino acids like histidine. In support of this, we previously showed by fluorescent microscopic analysis that *B*. *melitensis* host cells in the spleen of IL-12^-/-^ BALB/c mice are particularly lipid-rich [23]. It is not known whether *B*. *melitensis* has access to large quantities of glutamine within its replication niche *in vivo*. However, glutamine is an amino acid that is very abundant in mammals and fuels many tissues as well as immune system cells [56]. It is one of the most abundant amino acids in the THP-1 macrophage cell line [57]. Glutamine is a favored source of nitrogen and, for example, is required for adenine biosynthesis via the PurA,B,C,E,H,Q,S enzymes. Glutamine can be also used to produce glutamate via GOGAT [42] or, possibly, via a glutaminase *purQ* (BMEI1124). The latter was functionally identified as a glutaminase in *Bacillus subtilis* [58]. Glutamate can be consumed by the TCA cycle to produce pyruvate and ultimately glucose (Fig 6). In addition, in several bacteria, glutamate metabolism has been implicated in the resistance of bacteria to acid stress [59]. The glutamate decarboxylase (GAD) system facilitates intracellular pH homoeostasis by consuming protons in a decarboxylation reaction that produces c-aminobutyrate (GABA) from glutamate. However, *B*. *melitensis* strains do not have a functional GAD system [60].

Comparison of our Tn-seq data obtained in the lungs and spleen demonstrates that several sets of genes are specifically required for *B*. *melitensis* in the lungs or spleen. Among the 12 genes predicted to be essential in the lungs and not in the spleen, a clustering analysis identified a cluster involved in tryptophan synthesis, a cluster associated with polymyxin resistance, and a cluster associated with respiration that are indispensable in the lungs and not in the spleen (Fig 3A). It is well described that the innate immune response depletes cellular tryptophan in response to infection via the host enzyme indoleamine 2,3-dioxygenase (IDO-1) that converts tryptophan to N-formylkynurenine, which is a potent negative regulator of inflammation. The tryptophan biosynthetic pathway has been shown to be essential for host colonization by *Mycobacterium tuberculosis*. Δ*trpD M*. *tuberculosis* failed to cause disease in both wild-type and severe combined immune-deficient (SCID) mice [61]. The *bveA* (BMEII0681) gene of *B*. *melitensis* encodes a phospholipase A1 with specificity for phosphatidylethanolamine (PE). By reducing the level of PE in the bacterial membrane, *bveA* increases the resistance of *B*. *melitensis* to polymyxin and is required for persistent infection in mice [62]. To our knowledge, the importance of *ctaA* (BMEI1172), a heme A synthase, and *ctaG* (BMEI1463), a cytochrome c oxidase assembly protein, in mice has not been described.

Among the 34 genes predicted to be essential in the spleen and not in the lungs, a clustering analysis identified only four small clusters (Fig 3B): one cluster associated with amino acid biosynthesis, one identified as a zinc transporter, one identified as an efflux transporter and one identified as an ABC transporter. The other 23 genes could not be linked to a gene cluster and 5 of them are unidentified. Zinc (Zn^2+^) is an essential metal required by bacteria as either a structural or catalytic cofactor but free Zn^2+^ concentrations in mammalian hosts are very low. The *znuABC* operon constitutes a high-affinity periplasmic binding protein-dependent ATP-binding cassette (ABC) transport system used by bacteria for the uptake of Zn^2+^. It has been reported that Δ*znuA B*. *melitensis* [63] and Δ*znuA B*. *abortus* [64] are attenuated in the spleen of BALB/c mice.

Taken together, these results suggest that *B*. *melitensis* faces different nutritional conditions in the lungs and spleen. In particular, *B*. *melitensis* is thought to face deprivation of tryptophan in the lungs and of Zn^2+^ in the spleen. We confirmed using deletion mutants that the *trpD* (BMEI0844) gene is essential in the lungs and not in the spleen and that the *znuA* (BMEII0178) gene is essential in the spleen and not in the lungs.

The fact that persistence of *B*. *melitensis* in the lungs and spleen requires specific sets of genes suggests that it should be possible to use our Tn-seq data to develop vaccine candidates capable of persisting in organs long enough to induce development of a protective immune memory but unable to colonize the reservoir of the spleen on a long-term basis. Using these criteria (summarized in Fig 9), we selected two candidate genes, *murI* (BMEI0795) and *plsC* (BMEI1977). We predicted that inactivation of *murI* would induce very strong attenuation of *B*. *melitensis* in the spleen and that inactivation of *plsC* would result in moderate attenuation. We constructed deletion mutants of these genes and validated our Tn-seq predictions.

To our knowledge, *murI* and *plsC* have not been characterized in *Brucella* spp. The *murI* gene is predicted to code for a glutamate racemase, an enzyme involved in peptidoglycan (PG) biosynthesis. By converting L-glutamate to D-glutamate, this enzyme participates in the synthesis of peptide stems of PG, and thus contributes more generally to the integrity and growth of the bacteria [65]. In the literature, the *murI* gene is known to be the only enzyme that is able to synthesize D-Glu in *E*. *coli* [66] and *M*. *tuberculosis* [67] and is essential for their growth *in vitro*. The *plsC* gene coding for the integral membrane protein PlsC, an Acyl-sn-glycerol-3-phosphate acyltransferase. In mammals, PlsC enzyme is involved in phospholipid biosynthesis and is therefore required for epidermal permeability barrier homeostasis [68]. It could be hypothesized that inactivation of *plsC* in *B*. *melitensis* might change the properties of inner and outer membrane, potentially having pleiotropic effects on mechanisms such as effector secretion, resistance to antimicrobial peptides or alterations in the ability of the bacterium to adapt to environmental changes such as variations in acidity and osmolarity. Identifying the mechanisms explaining attenuation of Δ*plsC B*. *melitensis* strain in mice is difficult work that is beyond the scope of our study.

We compared the ability of deletion mutants for the *murI* and *plsC* genes to induce protective immunity in wild-type C57BL/6 mice. Surprisingly, we observed that the strongly attenuated Δ*murI* strain induces only weak immunity in the lungs and spleen (-1 log CFU) whereas the moderately attenuated Δ*plsC* strain induces immunity similar to the wild-type and Rev.1 strains. These results suggest that it is essential for the vaccine strain to be able to multiply in the lungs and the spleen to induce the development of protective immunity in these organs. Excessive attenuation prevents activation of the immune system. This suggests that the activation of adaptive immunity by *B*. *melitensis* indeed requires the completion of an infection cycle, and that the simple administration of antigens is ineffective in protecting the animal. These results can be correlated with the induction of specific *Brucella* IgG2a antibodies. Protective wild type, Δ*plsC* and Rev.1 strains induce high levels of antibodies while non-protective strains such as Δ*virB* and Δ*murI* strains do not. Very interestingly, we observed that persistence of the Δ*plsC* strain in mice at 28 days post-vaccination was lower than that of the Rev.1 strain, suggesting that the Δ*plsC* strain may be safer than the Rev.1 strain.

Our approach has several limitations. First, *B*. *melitensis* can infect and persist in many organs *in vivo* and we only analyzed two of them in C57BL/6 mice. A more extensive functional map, including other organs of interest, such as the lymph nodes and placenta, would be useful to better understand the biology of *B*. *melitensis* in the mouse model. Secondly, it is not advisable to make attenuation of a vaccine dependent on the deletion of a single gene. Consequently, it would be useful to carry out new Tn-Seq analyses in the organs of interest using a library constructed from the Δ*plsC* strain and to compare these results to those already available with the library constructed from the wild-type strain. This approach would identify other genes that can be deleted without the risk of excessively reducing the virulence of the vaccine candidate in the spleen, while guaranteeing the desired level of attenuation. Thirdly, since mice are not the natural host of *B*. *melitensis* and in light of the fact that many vaccines developed in mice have failed to induce immunity in cattle, Tn-seq analyses should be carried out in the spleen of goats or sheep in order to validate the results obtained in mice.

Overall, our results demonstrate that *B*. *melitensis* faces very different environments *in vivo* depending on the organs infected. Identifying the nutritional requirements of *B*. *melitensis in vivo* may open up new avenues for brucellosis treatment. For example, tryptophan synthase inhibitors from *M*. *tuberculosis* have been developed and have been shown to be effective in blocking its growth [69]. Our results also show that the construction of a functional map of the *B*. *melitensis* genome using Tn-seq analyses carried out on different organs can be a valuable aid for the development of effective attenuated vaccine candidates in mice, with a safer profile.

## Materials and methods

### Ethics statement

The procedures used in this study and the handling of the mice complied with current European legislation (Directive 86/609/EEC). The Animal Welfare Committee of the Université de Namur (UNamur, Belgium) reviewed and approved the complete protocol for *Brucella melitensis* infection (Permit Number: UN-LE-18/309 and UN-LE-23/401).

### Mice and bacterial strains

Wild-type C57BL/6 mice were acquired from Harlan (Bicester, UK). IFN-γR^-/-^ C57BL/6 mice [17] were acquired from Dr B. Ryffel (University of Orleans, France). All wild-type and deficient mice used in this study were bred in the animal facility of the Gosselies campus of the Université Libre de Bruxelles (ULB, Belgium).

The wild-type *B*. *melitensis* 16M strain used here is a Nal^R^ derivative of wild-type *B*. *melitensis* 16M [70]. We also used wild-type [22] and Δ*virB*1-12 (BMEII0025-35) [71] *B*. *melitensis* 16M strains stably expressing the mCherry fluorescent protein under the control of the strong *Brucella* spp. promoter p_*secE*_, also called p_sog_ or p_*sojA*_ [72]. *B*. *melitensis* Rev.1 is the World Organisation for Animal Health (WOAH) recommended goat and sheep brucellosis vaccine and was obtained from Sciensano (Belgium).

*Brucella melitensis* was always handled in BSL-3 containment facilities according to Council Directive 98/81/EC of 26 October 1998 and the law of the Walloon government of 4 July 2002.

### Transposon mutagenesis

One milliliter of an overnight culture of a nalidixic acid-resistant strain of *B*. *melitensis* 16M was mixed with 50 μL of an overnight culture of the conjugative *Escherichia coli* S17-1 strain carrying the pXMCS-2 mini-Tn*5* Kan^r^ plasmid [73]. This plasmid possesses a hyperactive Tn*5* transposase allowing for straightforward generation of a high number of Tn mutants, as described previously [73]. The mating mixture was incubated overnight at room temperature (RT) on 2YT agar plates (rich medium, 1% yeast extract, 1.6% peptone, 0.5% NaCl, 2% agar). The resulting *B*. *melitensis* Tn mutants were selected on 2YT agar plates supplemented with both kanamycin (10 μg/mL) and nalidixic acid (25 μg/mL). Tn*5* mutagenesis generates insertion of the transposon at only one locus per genome, as demonstrated previously for *Brucella* [74].

### Analysis of essential genes for growth on plates

Genomic DNA was extracted from a spleen transposon library using standard techniques and prepared for transposon library sequencing. Briefly, *B*. *melitensis* Tn mutants from each plate were collected, mixed and killed by heat (1 hour, 80°C). The lysate was incubated with a mixture composed of Tris (tris-hydroxymethyl-aminomethane 50 mM), EDTA (Ethylenediaminetetraacetic acid, 50 mM), 0.1 M NaCl, Proteinase K (20 mg/mL) and 10% SDS. The mixture was treated with an equal volume of 100% isopropanol to precipitate the DNA, which was washed with 70% ethanol. Genomic DNA was resuspended in deionized water and genomic DNA flanking the Tn*5* was sequenced (Fasteris company, Geneva, Switzerland). Libraries were sequenced on an **Illumina HiSeq** (for spleen from IFNγR^-/-^ mice conditions) or on an **Illumina NextSeq** (for 2YT, lung and spleen from wild-type mice conditions) with a primer hybridized at the border of the transposon, with its 3’ end pointing toward the flanking genomic DNA. Raw reads for each biological conditions (available at https://doi.org/10.6084/m9.figshare.26063158.v1) were mapped on *B*. *melitensis* 16M (accession numbers NC_003317 and NC_003318 for chromosomes I and II, respectively) using BWA [75] and read counts were determined using the samtools suite [76]. To account for truncated but functional products and misannotated start sites, only insertions in the central 80% of each gene were considered.

To determine if an insertional mutant in a defined gene is affected in a condition but untouched in the control 2YT condition, each gene was assigned an insertion index, called the **transposon insertion frequency** (**TnIF**), equal to the log_10_ of its total read count (thus including multiple insertions at the same position) +1, divided by its length (in bp), corresponding to 80% of the internal segment of the coding sequence. For each gene, a ΔTnIF (TnIF_cdt_−TnIF_CTRL_) value was calculated, where TnIF was computed for the tested condition (TnIF_cdt_) and the control condition (TnIF_CTRL_). The frequency distribution of ΔTnIF values was plotted for both chromosomes and for each condition tested (S3 Fig), to identify the main peak of unaffected ΔTnIF values and its standard deviation. 2% of ΔTnIF values at each extremity were removed to avoid an influence of extreme values. The standard deviation was calculated on this distribution. Depending on the conditions tested, the standard deviation ranged from 0.19 and 0.30 with a mean of 0.24. The ΔTnIF values greater than 0.5 were thus selected as significant, since they correspond to 1.6 to 2.6 standard deviations from the mode, designating genes for which the TnIF value was decreased compared to the control condition.

### Construction of deletion mutants to test Tn-seq predictions

Construction of the Δ***trpD*** (BMEI0843) and Δ***gmd*** (BMEI1413) *B*. *melitensis* 16M strains has been previously described [16]. The Δ***lysR*** (BMEI0513), Δ***murI*** (BMEI0795), Δ***plsC*** (BMEI1977), Δ***purH*** (BMEI0233) and Δ***znuA*** (BMEII0178) deletion strains were constructed in the *B*. *melitensis* 16M wt strain by triparental mating to introduce the pNPTS138 Kan^R^ plasmid (containing the upstream joined to the downstream region, generated by PCR, for the respective genes of interest for deletion) in the *B*. *melitensis* 16M Nal strain using the *E*. *coli* MT 607 (*pro-82 thi-I hsdR17* (r-m+) *supE44 recA56* pRK600) strain (as described in [77]), and allelic replacement was performed as described previously for other gene deletions [78]. These mutant strains do not have inserted antibiotic resistance genes.

Δ*gmd*, Δ*lysR*, Δ*murI*, Δ*plsC*, Δ*purH*, Δ*trpD* and Δ*znuA* deletion strains were conjugated with *E*. *coli* S17-1 containing the pSK kanR DsRed plasmid to introduce genes for kanamycin resistance and to express DsRed. Deletion of the genes was checked using the respective primers: the lysR-CheckF and lysR-CheckR primers for *lysR*, the murI-CheckF and murI-CheckR primers for *murI*, the plsC-CheckF and plsC-CheckR primers for *plsC*, purH-CheckF and purH-CheckF primers for *purH*, the znuA-CheckF and znuA-CheckR primers for *znuA*. See S3 Table for primer sequences used to amplify upstream and downstream regions of each gene.

The Δ*murI* and Δ*plsC* deletion mutants were complemented with the pMR10 vector containing the gene corresponding amplified with primers, as listed in S3 Table, under the control of the p_*lac*_ promoter (pMR10::*trpD*, pMR10::*murI*).

### *Brucella melitensis* infection in vivo

Mice were anesthetized with a cocktail of Xylazine (9 mg/kg) and Ketamine (36 mg/kg) in PBS before being inoculated by intranasal injection with 10^5^ or 5 × 10^6^ CFU of *B*. *melitensis* in 30 μL of RPMI, as indicated. Intraperitoneal injection of 5 × 10^6^ CFU of *B*. *melitensis* in 500 μL of RPMI was performed without anesthesia as described previously [27]. Control animals were inoculated with the same volume of RPMI. We used a mCherry-expressing wild-type (wt) 16M strain [79], mCherry-expressing Δ*virB* 16M strain [22] or DsRed-expressing gene deletion mutants in the wild-type *B*. *melitensis* 16M background as indicated for the infections. Cultures were grown overnight with shaking at 37°C in 2YT medium and were washed twice in RPMI 1640 (Gibco Laboratories) (3500 g, 10 min) before inoculation in the mice. The infectious doses were validated by plating serial dilutions of the inoculums. At the selected times after infection, mice were sacrificed by cervical dislocation. Immediately after sacrifice, spleen and/or lung cells were collected for bacterial counting or bacterial DNA extraction. All infections were performed in an Animal Biosafety Level 3 facility.

For bacterial counting, organs were homogenized in PBS/0.1% X-100 Triton (Sigma-Aldrich). We performed successive serial dilutions in RPMI to obtain the most accurate bacterial count and plated them on 2YT medium. The CFU were counted after 4 days of incubation at 37°C.

### Protocol for secondary infection with *Brucella melitensis*

C57BL/6 mice were immunized intranasally (i.n.) with 10^5^ CFU of live wild-type or deletion mutants of *B*. *melitensi*s 16M, as indicated. The infectious doses were validated by plating serial dilutions of inoculums. Six weeks after immunization, the mice were challenged i.n. with 10^5^ CFU of live wild-type mCherry-*B*. *melitensis* and sacrificed at 12 days post-infection. It is important to note that none of the strains used for vaccination has Kan resistance or expresses a fluorochrome. The expression of Kan resistance and the mCherry fluorochrome by the challenge strain makes it possible to easily differentiate the vaccine strains from the challenge strain when counting the bacterial colonies on plates of 2YT-Agar medium with or without kanamycin (50 μg/mL) since bacteria with mCherry form pink colonies on plates.

### Enzyme-linked immunosorbent assay (ELISA)

The presence of *Brucella melitensis* specific murine IgG2a was determined by ELISA. Polystyrene plates (269620; Nunc) were coated with Heat Killed (HK) *B*. *melitensis* (10^7^ CFU/mL) and incubated overnight at 4°C. The plates were blocked for 2 hours at RT with 200 μL/well of PBS-1% Bovine Serum Albumin (BSA). Then, plates were incubated with 50 μL/well of plasma in serial dilutions in PBS-0.1% BSA. The plasma of uninfected mice and PBS were used as negative controls. After four washes with PBS, isotype-specific goat anti-mouse HRP-conjugated Ab were added (50 μL/well) at appropriated dilutions (LO-MG2a-9 HRPO from LOIMEX). After 1 hour of incubation at RT, plates were washed four times in PBS and 100 μL/well of TMB substrate solution (BD OptEiA Kit) was added. After 15 minutes of incubation at RT in the dark, the enzyme reaction was stopped by adding 25 μL/well of 2 N H_2_SO_4_. The absorbance was measured at 450 nm.

### Statistical analysis

We used a (Wilcoxon-)Mann-Whitney test provided by the GraphPad Prism software to statistically analyze our results. Each group of deficient mice was compared with the wild-type mice. We also compared each group with the other groups and displayed the results when required. Values of p < 0.05 were considered to represent a significant difference. *, **, *** denote p < 0.05, p < 0.01, p < 0.001, respectively.

## Supporting information

S1 Fig Complementation of Δ*murI* and Δ*plsC* in the lungs and spleen. Data shown are bacterial counts (CFU) at the 120 hours post-infection in the lungs (**A**) and spleen (**B**) from wild-type mice infected intranasally (**A**) or intraperitoneally (**B**) with wild-type (wt), Δ*murI* or Δ*murI*-complemented, Δ*plsC* or Δ*plsC*-complemented strains of *B*. *melitensis* at a dose of 5x10^6^ CFU. Red lines represent the geometric mean. Dotted lines represent the mean of the wild-type strain. Significant differences between wt and the indicated groups are marked with asterisks: *p < 0.1, **p < 0.01, ***p < 0.001, ****p < 0.0001, in a (Wilcoxon-)Mann-Whitney post-test. These results are representative of two independent experiments.
(TIF)

S2 Fig Humoral immune response induced by intranasal *B*. *melitensis* strains infection. Wild-type C57BL/6 mice were infected intranasally with a dose of 10^5^ CFU of several strains of *B*. *melitensis* (wild-type (WT), Δ*plsC*, Rev.1, Δ*murI* or Δ*virB*). Sera were collected at 5 weeks post-infection, and ELISA was performed to determine the isotype distribution of the IgG2a *Brucella*-specific antibodies. The data represent the means ± SD of the results. O.D, optical density. These results are representative of two independent experiments.
(TIF)

S3 Fig Frequency distribution of ΔTnIF values of genes from chromosomes I and II of *B*. *melitensis* for each tested *in vivo* condition. For each condition (lungs, spleen, spleen from IFN-γ^-/-^), the ΔTnIF values are represented by classes of 0.2. The blue histogram shows the distribution for ΔTnIF values for all genes that are untouched in the control 2YT condition. The red color represents the distribution for ΔTnIF values without 2% of the genes at each extremity. SD = standard deviation.
(PDF)

S1 Table List of attenuated *B*. *melitensis* genes *in vitro* and in mice conditions. **Page 1**: List of the 3369 genes of *B*. *melitensis*. Each gene is associated with the value of TnIF in the 2YT, lungs and spleen conditions as well as the value of ΔTnIF associated with the lungs and spleen conditions. **Page 2**: List of 2460 genes that are not predicted to induce a growth defect in 2YT (TnIF > 2.7 in 2YT condition). **Page 3**: List of 135 genes predicted to cause attenuation in the lungs of wild type mice (ΔTnIF < -0.5 in lungs condition). **Page 4**: List of 257 genes predicted to cause attenuation in the spleen of wild type mice (ΔTnIF < -0.5 in spleen condition). **Page 7**: List of the 4 genes selected as vaccine candidates showing strong attenuation in the spleen (dTnIF < -2.0 in spleen condition). **Page 8**: List of the 17 genes selected as vaccine candidates showing moderate attenuation in the spleen (dTnIF < -1.0).
(XLSX)

S2 Table List of *B*. *melitensis* genes that are predicted as attenuated in the spleen of wild-type mice but that are predicted as not attenuated in the spleen of IFNγR^-/-^ mice. Bacterial genes required to grow in the spleen of wild-type (wt) mice infected intraperitoneally (ΔTnIF < -1.0) and that are not attenuated in the spleen of IFNγR^-/-^ mice infected by the same route (ΔTnIF > -0.5). NA = not assigned.
(DOCX)

S3 Table List of primers used in the construction of deletion mutants and for complementation of mutants. (DOCX)
