# Supplementary figures and images for: Genome-wide analysis of Brucella melitensis growth in spleen of infected mice allows rational selection of new vaccine candidates
Source: PLoS Pathog. 2024 Aug 26;20(8):e1012459. doi: 10.1371/journal.ppat.1012459 (PMC11346958; doi:10.1371/journal.ppat.1012459)

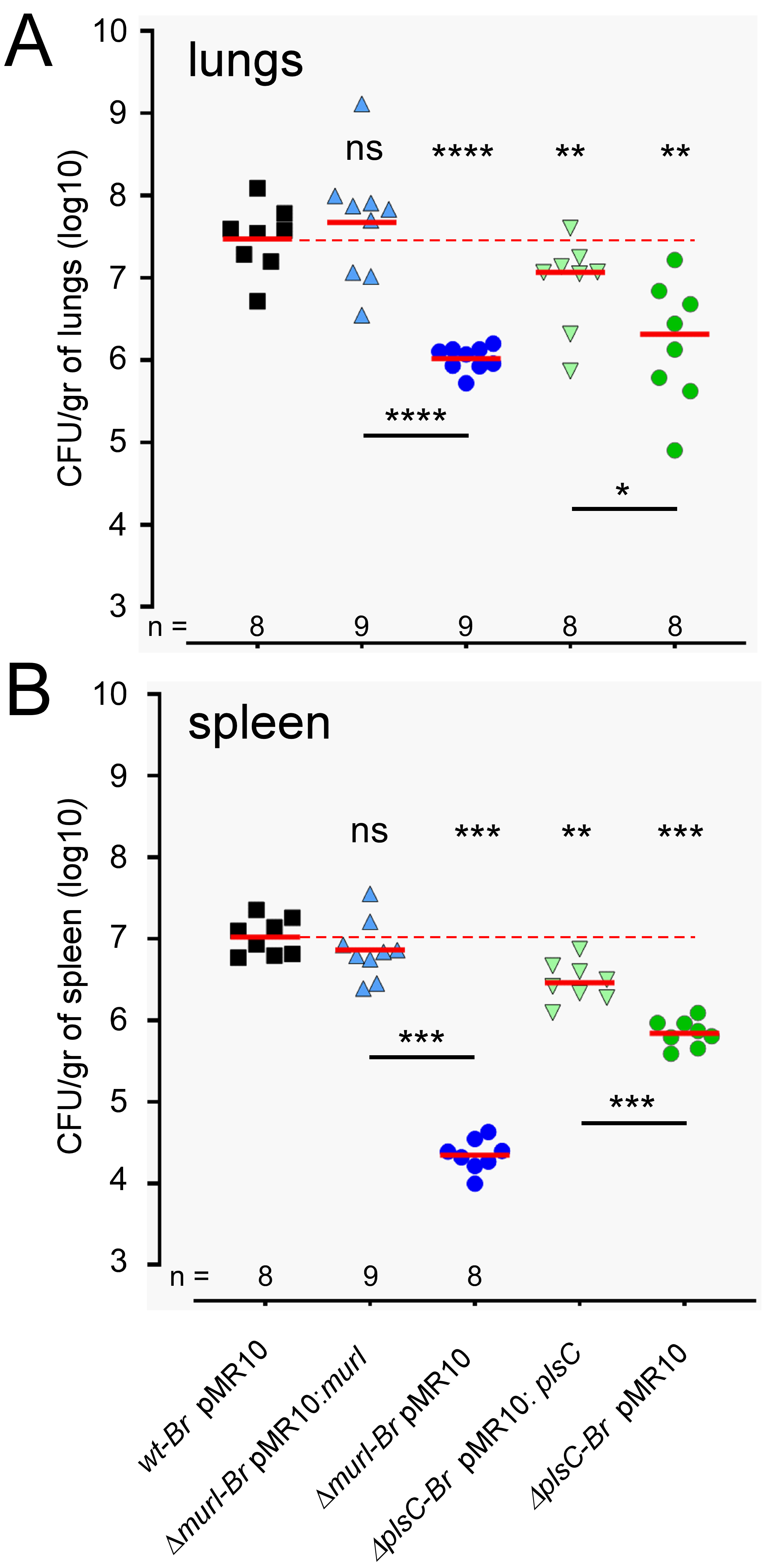

Supplement: S1 Fig — Data shown are bacterial counts (CFU) at the 120 hours post-infection in the lungs (A) and spleen (B) from wild-type mice infected intranasally (A) or intraperitoneally (B) with wild-type (wt), ΔmurI or ΔmurI-complemented, ΔplsC or ΔplsC-complemented strains of B. melitensis at a dose of 5x10^6^ CFU. Red lines represent the geometric mean. Dotted lines represent the mean of the wild-type strain. Significant differences between wt and the indicated groups are marked with asterisks: *p < 0.1, **p < 0.01, ***p < 0.001, ****p < 0.0001, in a (Wilcoxon-)Mann-Whitney post-test. These results are representative of two independent experiments. (TIF) [file ppat.1012459.s001.tif]

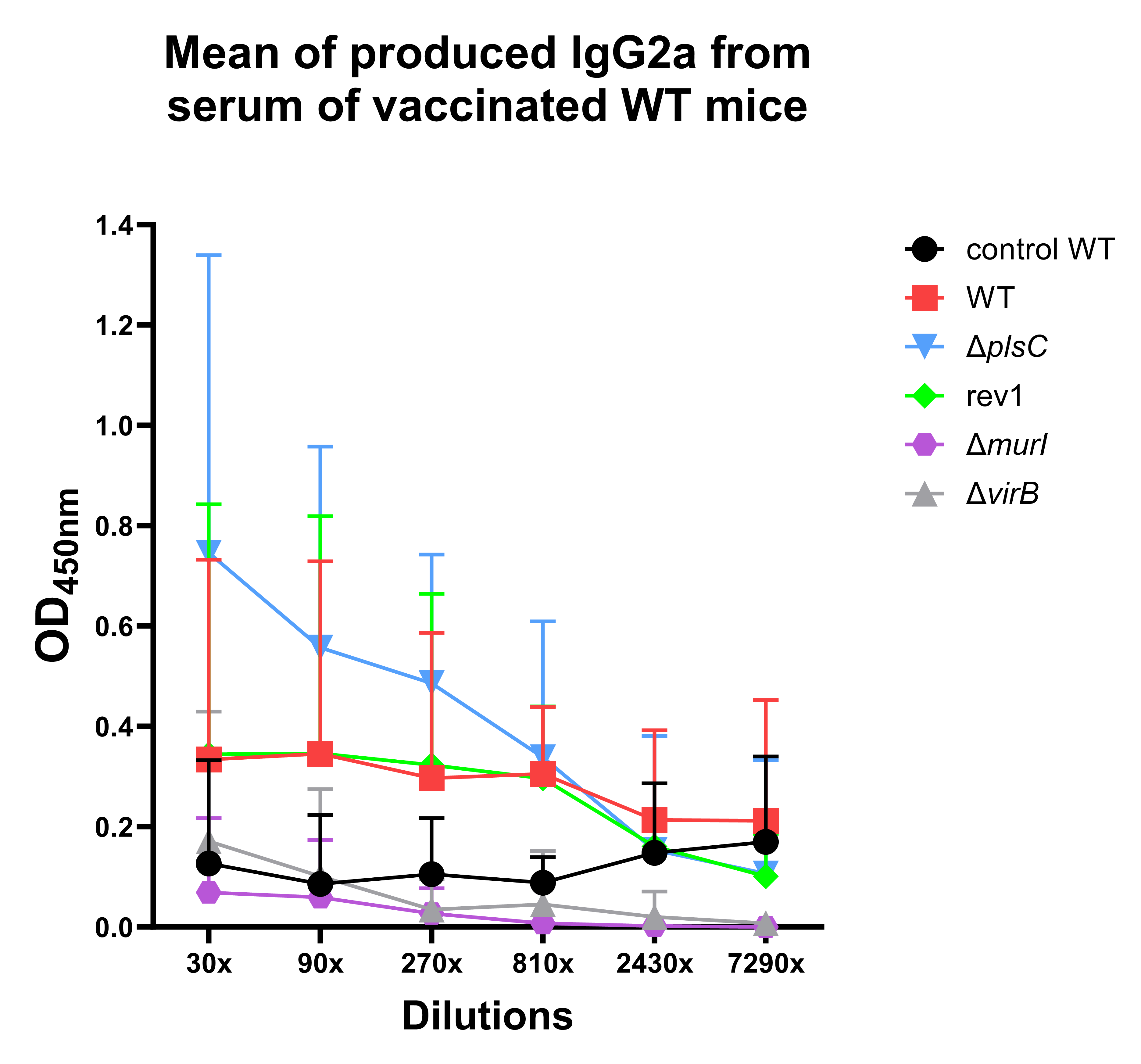

Supplement: S2 Fig — Wild-type C57BL/6 mice were infected intranasally with a dose of 105 CFU of several strains of B. melitensis (wild-type (WT), ΔplsC, Rev.1, ΔmurI or ΔvirB). Sera were collected at 5 weeks post-infection, and ELISA was performed to determine the isotype distribution of the IgG2a Brucella-specific antibodies. The data represent the means ± SD of the results. O.D, optical density. These results are representative of two independent experiments. (TIF) [file ppat.1012459.s002.tif]

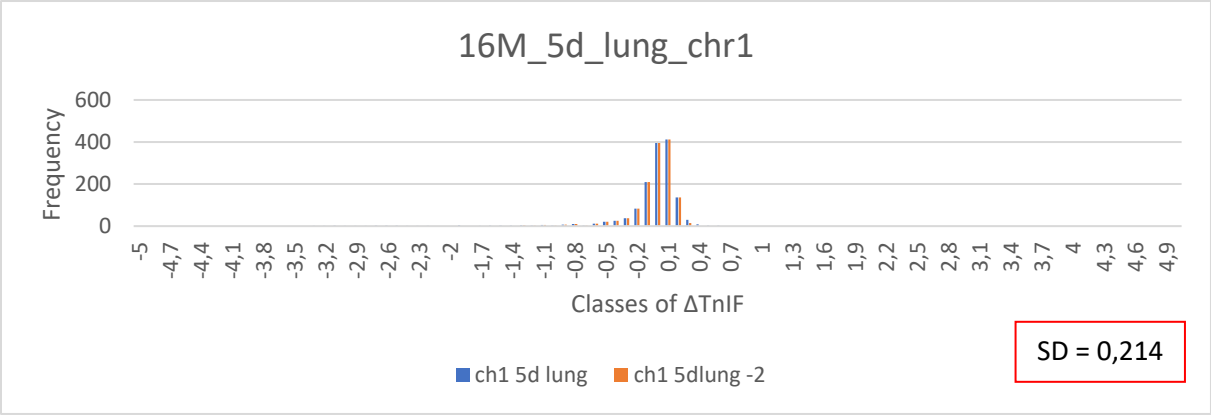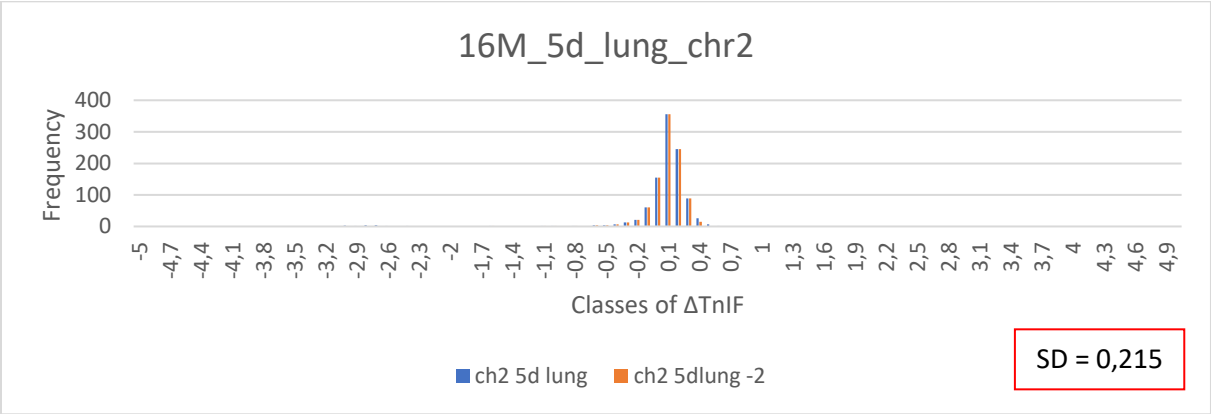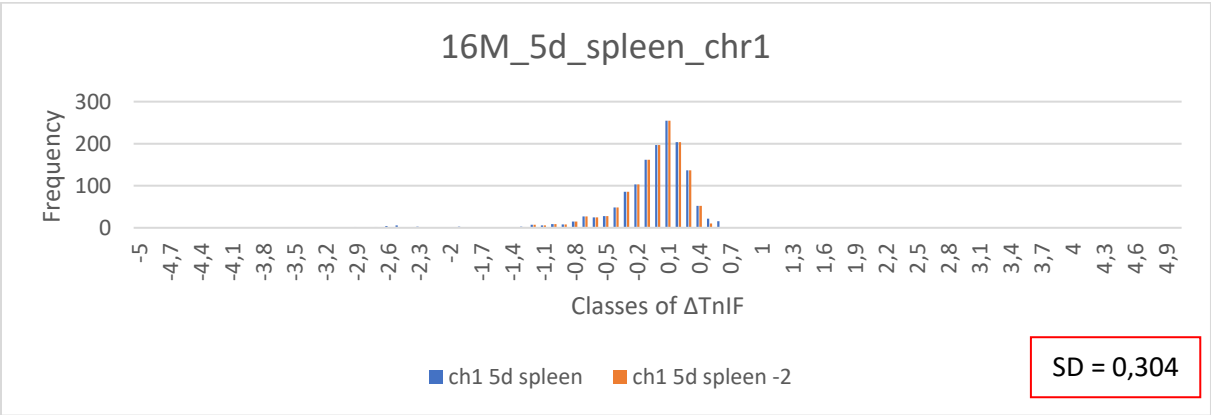

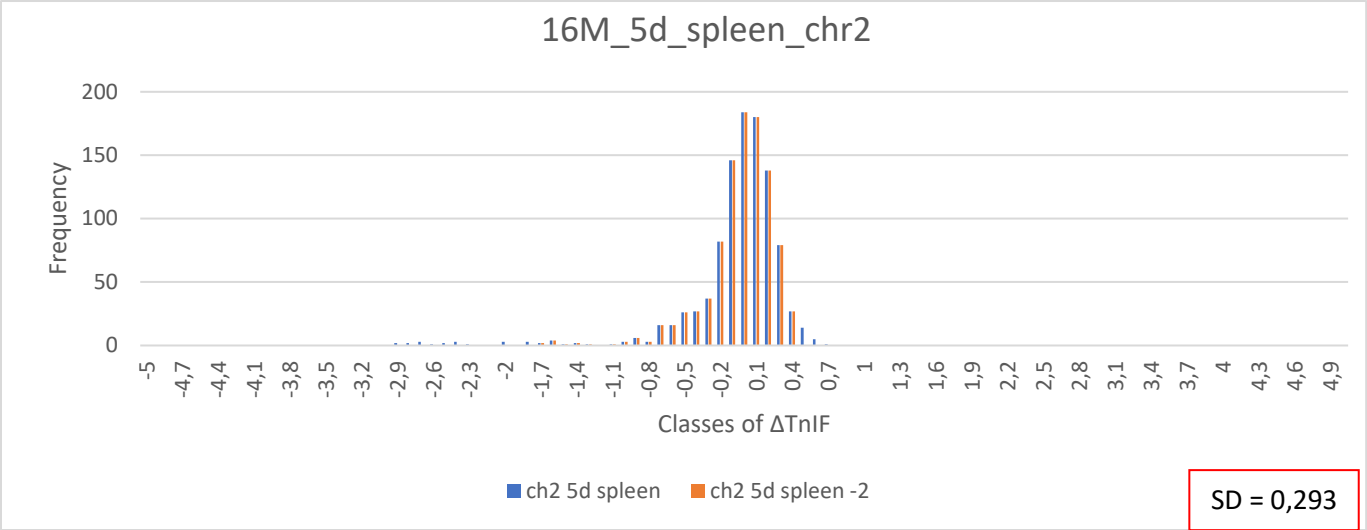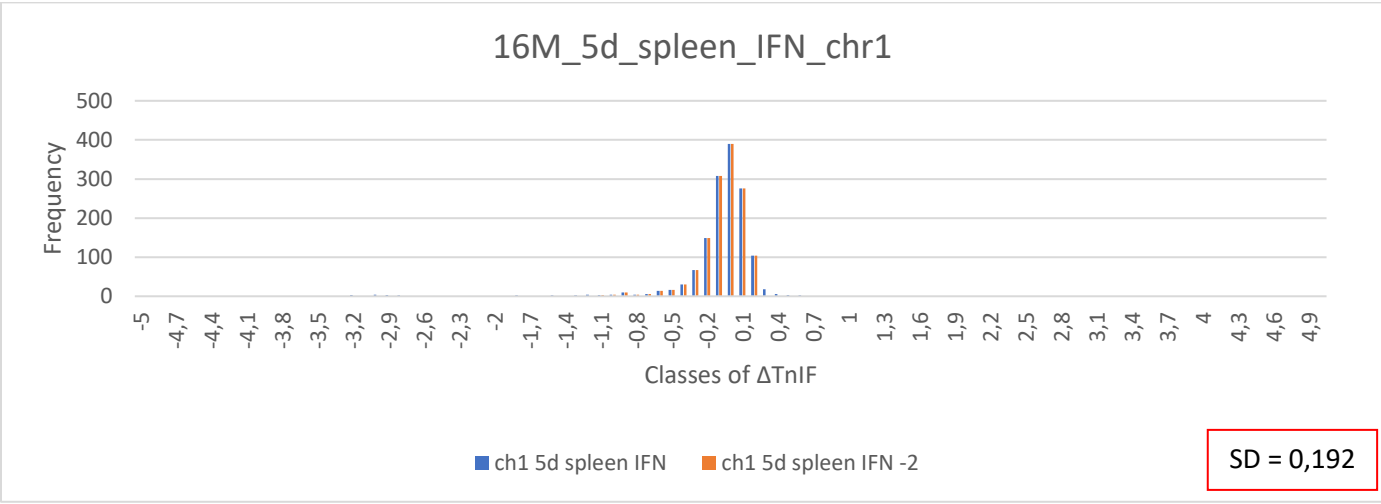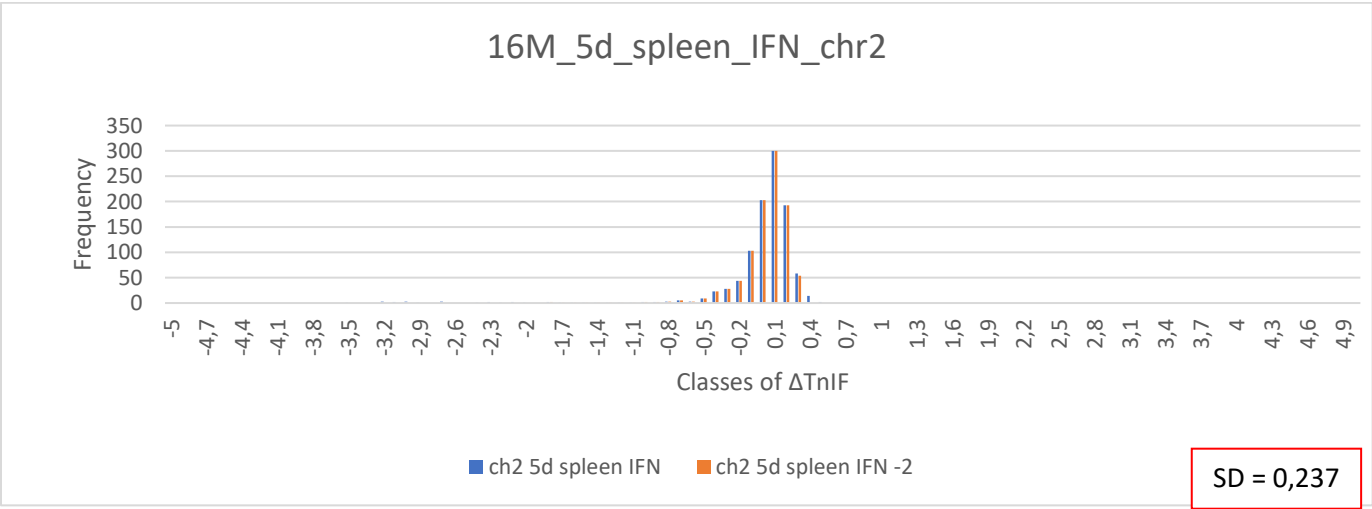

|                            | SD         |
|----------------------------|------------|
|                            | 0,21421628 |
|                            | 0,21463183 |
|                            | 0,30356721 |
|                            | 0,29347291 |
|                            | 0,19214614 |
|                            | 0,23667545 |
| Mean                       | 0,24245164 |
| 1SD=0,24                   |            |
| threshold of 0,5 = 2,08 SD |            |

Supplement: S3 Fig — For each condition (lungs, spleen, spleen from IFN-γ-/-), the ΔTnIF values are represented by classes of 0.2. The blue histogram shows the distribution for ΔTnIF values for all genes that are untouched in the control 2YT condition. The red color represents the distribution for ΔTnIF values without 2% of the genes at each extremity. SD = standard deviation. (PDF) [file ppat.1012459.s003.pdf]
