# Supplementary material for: Genome-wide analysis of Brucella melitensis growth in spleen of infected mice allows rational selection of new vaccine candidates
Source: PLoS Pathog. 2024 Aug 26;20(8):e1012459. doi: 10.1371/journal.ppat.1012459 (PMC11346958; doi:10.1371/journal.ppat.1012459)

**Table S2: List of *B. melitensis* genes that are predicted as attenuated in the spleen of wild-type mice but that are predicted as not attenuated in the spleen of IFNγR^-/-^ mice.** Bacterial genes required to grow in the spleen of wild-type (wt) mice infected intraperitoneally (∆TnIF < -1.0) and that are not attenuated in the spleen of IFNγR^-/-^ mice infected by the same route (∆TnIF > -0.5). NA = not assigned.


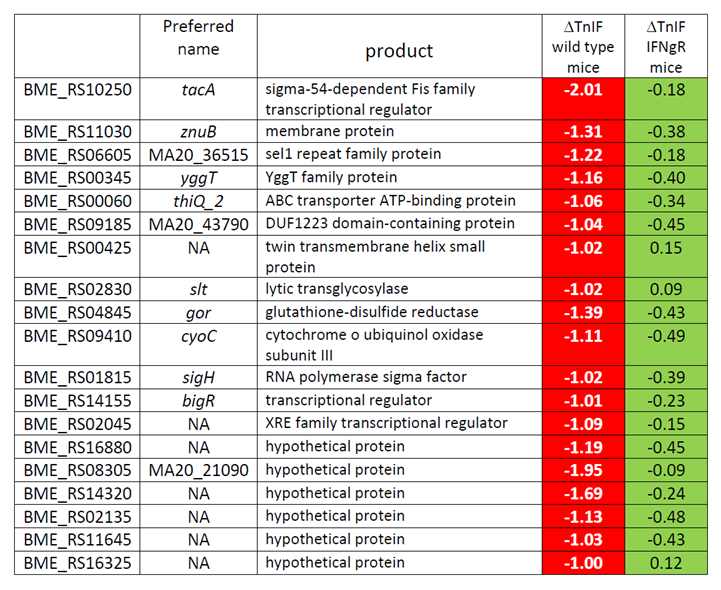

Supplement: S2 Table — Bacterial genes required to grow in the spleen of wild-type (wt) mice infected intraperitoneally (ΔTnIF < -1.0) and that are not attenuated in the spleen of IFNγR-/- mice infected by the same route (ΔTnIF > -0.5). NA = not assigned. (DOCX) [file ppat.1012459.s005.docx]
