# Supplementary material for: Genome-wide analysis of Brucella melitensis growth in spleen of infected mice allows rational selection of new vaccine candidates
Source: PLoS Pathog. 2024 Aug 26;20(8):e1012459. doi: 10.1371/journal.ppat.1012459 (PMC11346958; doi:10.1371/journal.ppat.1012459)
Supplement: S3 Table — (DOCX) [file ppat.1012459.s006.docx]

**Table S3: List of primers used in the construction of deletion mutants and for complementation of mutants**

| **BMEI1413 *gmd*** | |
| --- | --- |
| Forward_check *gmd* | AGCTAACTTGCTGGCATAAG |
| Reverse_check *gmd* | CAGGTCGAGTCGATCATG |
| Forward_upstream *gmd* | CTAGAACTAGTGTCATTCTGTAATTATACGCAAGC |
| Reverse_upstream *gmd* | AAGGAATGATCCATACATGG |
| Forward_downstream *gmd* | CCATGTATGGATCATTCCTTTTTTTGCCATGCCAACTG |
| Reverse_downstream *gmd* | TGCACGTCGACCGAAGCGGTTGTAACAGG |
| **BMEI0513 *lysR*** | |
| Forward_check *lysR* | AAAGAGTTACAAGCTGTGGC |
| Reverse_check *lysR* | ATGGCGATTTCGTCTTCAC |
| Forward_upstream *lysR* | AAAATGTCGGTGCGCAAATCG |
| Reverse_upstream_ *lysR* | TTTATCCCAGTCAAGCGGTGC |
| Forward_downstream *lysR* | GCACCGCTTGACTGGGATAAAAATTGGACCTATTGACGG |
| Reverse_downstream *lysR* | TCATCGAAACCGACAAAG |
| **BMEI0795 *murI*** | |
| Forward_check_*murI* | GCCACGATTTCATAGCTAATGC |
| Reverse_check *murI* | GCGAAATACCGTTCGAGATGG |
| Forward_upstream  *murI* | GCAATCATTCTGGTCAATCCA |
| Reverse_upstream *murI* | GCCTTTCTGGAGCGATGG |
| Forward_downstream *murI* | CCATCGCTCCAGAAAGGCCCCGACTATGCCATTCGC |
| Reverse_downstream *murI* | AGCGCACCATCTTTCAGC |
| Forward_KpnI amplification *murI* | CGGGGTACCTGAGGACAAGGCAGAATG |
| Reverse_BamHI amplification *murI* | CGCGGATCCTTAAAAACGCAGCCCAAAACC |
| **BMEI0233 *purH*** | |
| Forward_check_*purH* | CACCACCATCATTGCCGA |
| Reverse_check *purH* | GATGCGGCAAAGGGTGAG |
| Forward_upstream  *purH* | TCAATTCCGGCATCGACC |
| Reverse_upstream *purH* | CCGATGAAGATCGGGAGC |
| Forward_downstream *purH* | GCTCCCGATCTTCATCGGATCGCCATGGTCATGACG |
| Reverse_downstream *purH* | TTGAGCGAGTTGAAGGCG |
| **BMEI1977 *plsC*** |  |
| Forward_check *plsC* | TTCTTGGAACACAACTGAACC |
| Reverse_check *plsC* | AGAAAGTGCAGAACTTGTC |
| Forward_upstream *plsC* | AGCGTATCGCCATCAATATC |
| Reverse_upstream *plsC* | CCAGAAATATCCTGATCGTAC |
| Forward_downstream *plsC* | GTACGATCAGGATATTTCTGGAAAAGCCGAGCAGATTGTCAC |
| Reverse_downstream *plsC* | TCAAACTTGTCTTCAATGGCG |
| Forward_KpnI amplification *plsC* | CGGGGTACCATGATCGGTACGATCAGG |
| Reverse_BamHI amplification *plsC* | CGCGGATCCTCAAGCCTCCGGGATTTC |
| **BMEI0844 *trpD*** |  |
| Forward_check *trpD* | GCTCGCCACCAATATTGC |
| Reverse_check *trpD* | CGTCATGTTGTCGCATAAGG |
| Forward_upstream *trpD* | CTGCACGATTCCTATGAGCAG |
| Reverse_upstream *trpD* | CACATCCACCGCTTTTAGAAG |
| Forward_downstream *trpD* | CTTCTAAAAGCGGTGGATGTGGTCATTGCCGTTTCCAACGAC |
| Reverse_downstream *trpD* | GTCCTCATGCGTGAAGATGC |
| **BMEII0178 *znuA*** | |
| Forward_check_*znuA* | ATGGTGTTCATGGTCATGG |
| Reverse_check *znuA* | AACCATAAAGCTCCTTGTACC |
| Forward_upstream  *znuA* | TGGCAATCAGTTCGTAAAGTG |
| Reverse_upstream *znuA* | CATGGTATCTCTCTTGAGCAA |
| Forward_downstream *znuA* | TTGCTCAAGAGAGATACCATGTCGAAATAAATCAGGCGCTCT |
| Reverse_downstream *znuA* | TATCTTGTTCAACACCACGAC |
